# Umbilical mesenchymal stem cell-derived exosomes facilitate spinal cord functional recovery through the miR-199a-3p/145-5p-mediated NGF/TrkA signaling pathway in rats

**DOI:** 10.1186/s13287-021-02148-5

**Published:** 2021-02-12

**Authors:** Yang Wang, Xunwei Lai, Depeng Wu, Bin Liu, Nanxiang Wang, Limin Rong

**Affiliations:** 1 Department of Spine Surgery, The Third Affiliated Hospital of Sun Yat-sen University, No. 600 Tianhe Road, Tianhe District, Guangzhou, Guangdong Province China; 2 Guangdong Provincial Center for Engineering and Technology Research of Minimally Invasive Spine Surgery, No. 600 Tianhe Road, Tianhe District, Guangzhou, Guangdong Province China; 3 Guangdong Provincial Center for Quality Control of Minimally Invasive Spine Surgery, No. 600 Tianhe Road, Tianhe District, Guangzhou, Guangdong Province China

**Keywords:** Exosomes, Umbilical cord mesenchymal stem cells, Spinal cord injury, microRNAs, TrkA ubiquitination, PC12 cells

## Abstract

**Background:**

Although exosomes, as byproducts of human umbilical cord mesenchymal stem cells (hUC-MSCs), have been demonstrated to be an effective therapy for traumatic spinal cord injury (SCI), their mechanism of action remains unclear.

**Methods:**

We designed and performed this study to determine whether exosomes attenuate the lesion size of SCI by ameliorating neuronal injury induced by a secondary inflammatory storm and promoting neurite outgrowth. We determined the absolute levels of all exosomal miRNAs and investigated the potential mechanisms of action of miR-199a-3p/145-5p in inducing neurite outgrowth in vivo and in vitro.

**Results:**

miR-199a-3p/145-5p, which are relatively highly expressed miRNAs in exosomes, promoted PC12 cell differentiation suppressed by lipopolysaccharide (LPS) in vitro through modulation of the NGF/TrkA pathway. We also demonstrated that Cblb was a direct target of miR-199a-3p and that Cbl was a direct target of miR-145-5p. Cblb and Cbl gene knockdown resulted in significantly decreased TrkA ubiquitination levels, subsequently activating the NGF/TrkA downstream pathways Akt and Erk. Conversely, overexpression of Cblb and Cbl was associated with significantly increased TrkA ubiquitination level, subsequently inactivating the NGF/TrkA downstream pathways Akt and Erk. Western blot and coimmunoprecipitation assays confirmed the direct interaction between TrkA and Cblb and TrkA and Cbl. In an in vivo experiment, exosomal miR-199a-3p/145-5p was found to upregulate TrkA expression at the lesion site and also promote locomotor function in SCI rats.

**Conclusions:**

In summary, our study showed that exosomes transferring miR-199a-3p/145-5p into neurons in SCI rats affected TrkA ubiquitination and promoted the NGF/TrkA signaling pathway, indicating that hUC-MSC-derived exosomes may be a promising treatment strategy for SCI.

**Supplementary Information:**

The online version contains supplementary material available at 10.1186/s13287-021-02148-5.

## Introduction

Acute traumatic spinal cord injury (SCI) has a prevalence rate of 10–83 per million [1] worldwide each year and usually results in devastating functional loss below the level of the injured spinal cord. The pathophysiology of SCI consists of primary mechanical injury and a subsequent cascade of inflammation, ischemia, and secretion of cytotoxic substances that aggravates SCI damage [2–4].

Neurons play a central role in transducing biological signals [5], but they fail to receive and send electrical and chemical signals following injury. Even though SCI patients achieve certain recovery of locomotion [6, 7], injured axons rarely reconnect to the same extent as axons in the normal spinal cord, and patient motor function never returns to normal [8]. A failed neuronal reconnection of two severed axons mainly occurs for the following reasons [8]: (a) loss of intrinsic outgrowth capacity of neurons, (b) deterioration of the microenvironment, and (c) inhibition of fibrous scar and glial scar formation. Therefore, promoting neurite outgrowth and improving the microenvironment of the remaining neurons should be essential for locomotor recovery in SCI patients.

TrkA, a critical member of the receptor tyrosine kinase (RTK) family composed of TrkA, TrkB, TrkC, and p^75^NTR, can be phosphorylated and induced to form intracellular dimers in the structural domain by coupling with NGF, which subsequently initiates signaling cascades through phosphorylating Ras protein. Based on previous studies on the mechanism of action of NGF/TrkA, which is indispensable in the development and maturation of the nervous system [9]. Exosomes, important paracrine factors of stem cells, are nanosized particles with a diameter of 20 to 150 nm and consist of a lipid bilayer that encapsulates RNAs, DNAs, and soluble proteins [10, 11]. Currently, an increasing number of researchers have demonstrated that stem cell-derived exosomes share similar therapeutic effects as stem cells, such as inhibition of proinflammatory cytokines [12], inactivation of A1 astrocytes [13], and conversion of macrophages to the M2 phenotype [14].

Through preliminary experiment, we demonstrated that UC-MSC exosomes can help to stimulate the NGF/TrkA signaling pathway and promote the neuronal markers expression (NF-H, Neu-N, and β-tubulin-III) inhibited by LPS in PC12 cells. Subsequently, we conducted this study to confirm the specific mechanisms of exosomes in inducing neurite outgrowth in vivo and in vitro and the efficacy of exosomes in treating SCI.

## Materials and methods

### Ethical statement

Ethical approval for the experiment was obtained from the Ethics Committee of the Third Affiliated Hospital of Sun Yat-sen University, China (Ethic number: IACUC-F3-19-0401). All experiments were performed following relevant laws and guidelines and the institutional guidelines of the Third Affiliated Hospital of Sun Yat-sen University. This article does not involve any human studies.

### Animal experimental protocol

We purchased adult female Sprague-Dawley rats (6–7 weeks old), all specific pathogen-free (SPF), from Shanghai SLAC Laboratory Animal Co., Ltd. (Shanghai, China). All rats were housed in an SPF environment (temperature, 22 ± 1 °C; humidity, 65–70%) with free access to food and water, and five rats were assigned per cage. All animal experiments were approved by the animal ethics committee of the Third Affiliated Hospital of Sun Yat-sen University and in accordance with the guidelines included in the Guide for the Care and Use of Laboratory Animals published by the National Institutes of Health (Eighth Edition). In total, seventy-six rats were sacrificed by CO_2_ suffocation (detailed animal information is provided in Additional file 1).

### Exosome purification and characterization

Exosomes were isolated from the supernatants of passage 5 hUC-MSCs according to the Total Exosome Extraction Reagent (Invitrogen, Carlsbad, California, USA) instructions. Before being subjected to transmission electron microscopy (TEM; Tokyo, Japan) and the qNano nanoparticle analysis system (Izon, Christchurch, New Zealand), exosomes in suspension were filtered using a 220-nm filter and stained with 3% aqueous phosphotungstic acid. Exosomes were lysed with radioimmunoprecipitation assay (RIPA) buffer (Beyotime, Shanghai, China) supplemented with a protease and phosphatase-inhibitor cocktail (CST, Boston, MA, USA). The protein concentration of exosomes was quantified using the BCA Protein Assay Kit (Thermo Fisher, Boston, MA, USA). Primary antibodies against CD63 (Abcam, Cambridge, UK), CD9 (Abcam, Cambridge, UK), and β-actin (CST, Boston, MA, USA) were used to characterize exosomes (detailed methods and materials are provided in Additional file 2).

### Statistical analysis

Statistical analysis was performed, and graphs were generated by using Graph Pad Prism 8.0 (San Diego, CA, USA). All data are presented as the mean ± standard deviation (SD) and the range from min to max, and differences were analyzed using one-way analysis of variance (one-way ANOVA). *P* < 0.05 was considered statistically significant.

## Results

### Exosomal miRNA profile analyzed by absolute quantitative sequencing

**Fig. 1:**
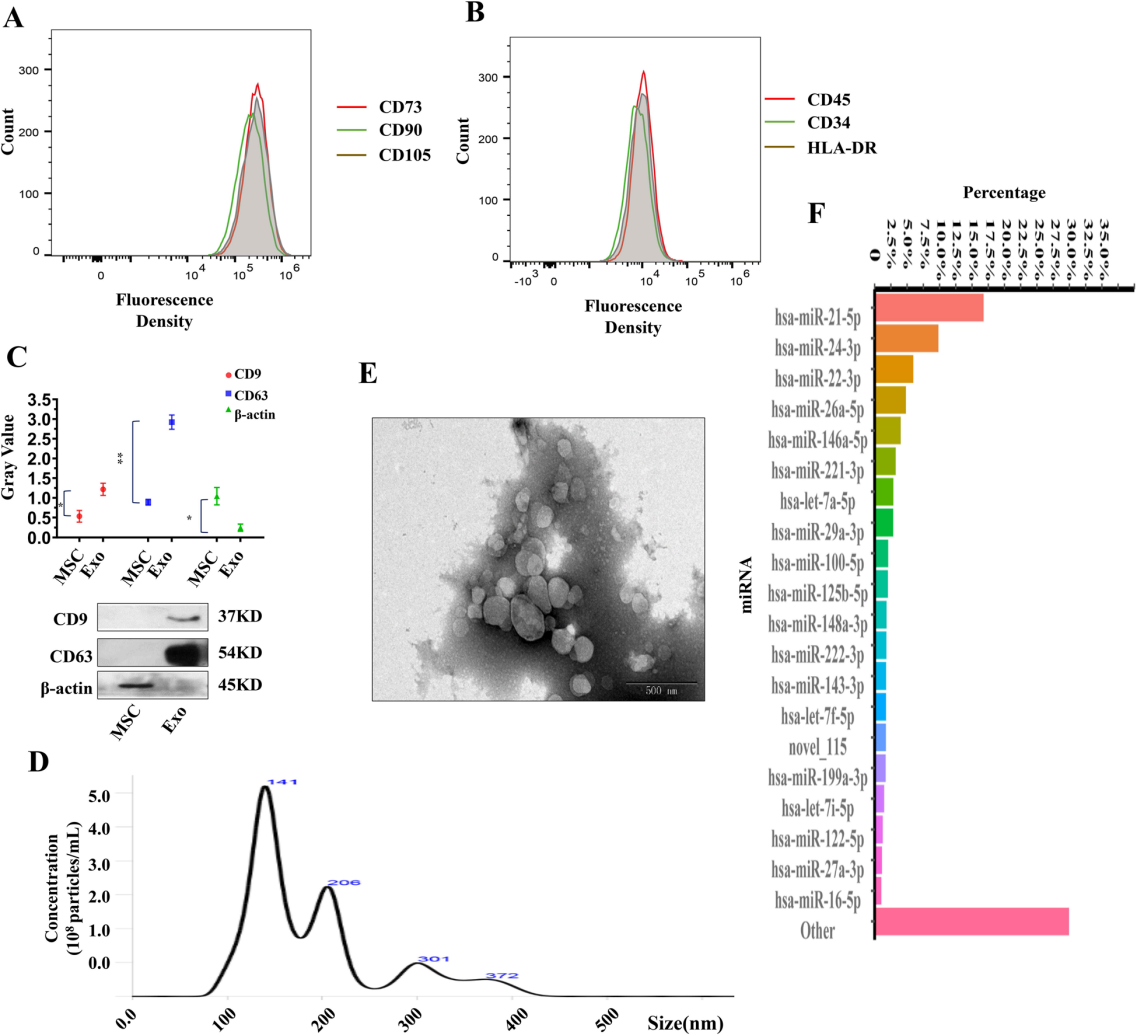
MSC and MSC-Exo characterization and exosomal miRNA profiles. hUC-MSCs were characterized by CD73, CD90, CD105 (**a**), CD45, CD34, and HLA-DR (**b**) expression by using flow cytometry. **c** Representative images and quantitative analysis of western blots to assess CD9, CD63, and β-actin expression in MSCs and MSC-Exo (*n* = 3). Data are represented as mean ± SD. **d** The morphology of exosomes detected by transmission electron microscopy (*n* = 3). Scale bar = 500 nm. **e** The diameters and concentrations of exosomes analyzed by the nanoparticle tracking method. **f** Top 20 miRNAs expressed in MSC-Exo. MSC mesenchymal stem cell, MSC-Exo MSC-derived exosomes. *p < 0.05; **p < 0.01

We obtained hUC-MSCs from Guangzhou Selera Stem Cell Technology Co., Ltd., and the cells that presented a long spindle shape were identified by flow cytometry. Stem cell markers (CD73, CD90, CD105) (Fig. 1a) were highly expressed, while non-stem cell markers (CD45, CD34, HLA-DR) (Fig. 1b) were expressed at extremely low levels on the cell surface. Then, we purified exosomes from the culture supernatants of MSCs by the Total Cell Exosome Extraction Kit. Analysis of the morphology and surface markers confirmed that the obtained pellet were exosomes. First, CD9 and CD63 were detected in these particles, which did not express β-actin (Fig. 1c). Nanoparticle tracking analysis (NTA) revealed the concentration and size of the particles ranging from 30 to 150 nm (Fig. 1d). Furthermore, a typical cup-shaped morphology of these nanoparticles was observed by transmission electron microscopy (TEM) (Fig. 1e).

miRNAs are the most abundant nucleic acids in MSC-derived exosomes (MSC-Exo) and play important roles in regulating cell activities. To obtain a better understanding of the exosomal miRNA profile, we performed small RNA absolute quantitative sequencing (see Additional file 6) of MSC-Exo, and the results are deposited in the GEO database of NCBI (GEO database: GSE 159814). The sequence results are provided in Additional file 6. Small RNA sequencing identified approximately 990 miRNAs in MSC-Exo. Further analysis showed a linear relationship between log-transformed standard deviation (SD) and transcripts per million (TPM) values (see Additional file 7A).

The top 20 miRNAs (plus miR-145-5p) and the miRNA-target network are provided (Fig. 1f, see Additional file 7B). miR-199a-3p (Fig. 1f) and miR-145-5p [15] were very abundant in MSC-Exo. We presumed that miR-199a-3p/145-5p regulate the TrkA turnover, because we found the direct relationship between miR-199a-3p and Cblb, miR-145-5p, and Cbl through on-line bioinformatic analysis (see Additional file 7B). As for Cbl and Cblb, they are critical enzyme in the modulation of TrkA degradation by lysosome. Subsequently, we examined how the exosomal miR-199a-3p/145-5p impact the neuronal differentiation through the regulation of Cblb and Cbl genes.

### miR-199a-3p/145-5p acted as critical effectors in MSC-Exo-mediated neuronal differentiation of PC12 cells in vitro and in vivo

**Fig. 2:**
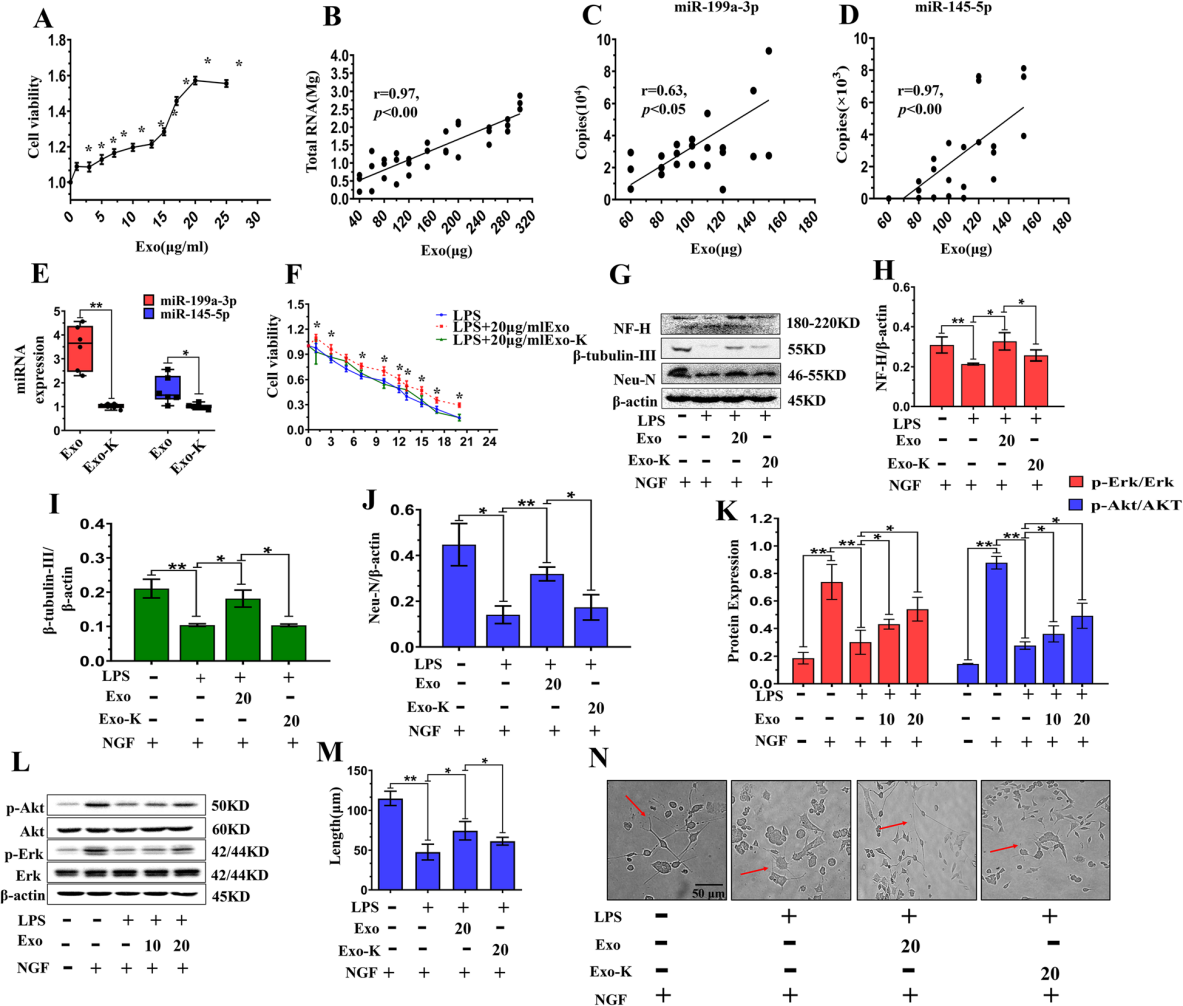
Exosomal miR-199a-3p/145-5p acted as critical effectors in the promotion of PC12 cell differentiation inhibited by LPS by regulating downstream factors of NGF/TrkA. **a** Viability of PC12 cells treated with increasing doses of MSC-Exo (*n* = 3). Data are represented as mean ± SD. **b** The linear relationship between total RNA and exosomal proteins. The linear relationship between miR-199a-3p (**c**), miR-145-5p (**d**), and exosomal proteins. **e** Relative miR-199a/145-5p expression in control MSC-Exo and MSC-Exo with the inhibition of miR-199a/145-5p (Exo-K) (*n* = 6). Data are represented from min to max. **f** Cell viability of LPS-treated PC12 cells pre-stimulated with Exo and Exo-K. Data are represented as mean ± SD. **g**–**j**)Representative western blot images and quantitative analysis of NF-H, β-tubulin-III, and Neu-N expression in LPS- and NGF-stimulated PC12 cells pretreated with Exo and Exo-K (*n* = 3). Data are represented as mean ± SD. (**k**-**l**) Representative western blot images quantitative analysis of p-Akt and p-Erk expression in LPS- and NGF-stimulated PC12 cells pretreated with different doses of exosomes (*n* = 3). Data are represented as mean ± SD. (**m**-**n**) Representative images and quantitative analysis of PC12 cell neurite growth (red arrow) in LPS- and NGF-stimulated PC12 cells treated with Exo and Exo-K. Scale bar = 50 μm. Data are represented as mean ± SD. *, *p* < 0.05; **, *p* < 0.01. LPS lipopolysaccharide, MSC mesenchymal stem cell, MSC-Exo MSC-derived exosomes, Exo-K MSC-Exo with miRNA inhibition

To investigate the synergistic effects of miR-199a-3p/145-5p in exosomes, we cotransfected MSCs with miR-199a-3p/145-5p antisense RNAs and then purified exosomes from the culture supernatants of nontreated MSCs (Exo) and cotransfected MSCs (Exo-K). First, we confirmed that PC12 cell viability increased dose-dependently with exosome concentrations (0, 1, 3, 5, 7, 10, 13, 15, 17, 20, 25 μg/ml), and 20 μg/ml was the most effective concentration in PC12 cells (1.6 times higher viability than that of the control) (Fig. 2a). The successful endocytosis of MSC-Exo by PC12 cells was confirmed by PKH26-labeled exosomes (See Additional file 8A). Subsequently, we confirmed that the total RNA content was positively associated with the amount of exosomal proteins (40, 60, 80, 100, 120, 150, 180, 200, 250, 280, 300 μg) quantified by BCA assay (Fig. 2b). Moreover, the levels of miR-199a-3p (Fig. 2c) and miR-145-5p (Fig. 2d) showed a linear relationship with the amount of exosomal proteins.

Interestingly, we found different miR-199a-3p/145-5p expression profiles between primary neurons, astrocytes, endothelial cells, and meningeal fibroblasts. Among these four types of primary cells, neurons had the highest levels of miR-145-5p and the lowest levels miR-199a-3p (see Additional file 8B). All primary cells were isolated from rats (see Additional file 8C). Moreover, through QRT-PCR analysis, we also discovered more miR-199a-3p transcripts than miR-145-5p transcripts in PC12 cells (See Additional file 8D).

Then, miR-199a-3p/145-5p expression was successfully inhibited in MSC-Exo (Exo-K) (Fig. 2e). Experiments were conducted to determine the optimal concentration of LPS, and the results demonstrated that the LPS concentration (0, 1, 3, 5, 7, 10, 11, 13, 15, 17, 20 μg/ml) had a dose-dependent effect on PC12 cell viability; 11 μg/ml LPS was selected to establish an injury model in PC12 cells (Fig. 2f). Furthermore, when PC12 cells were pretreated with 20 μg/ml MSC-Exo, cell viability was rescued (Fig. 2f). However, 20 μg/ml Exo-K could not rescue PC12 cell viability (Fig. 2f).

**Fig. 3:**
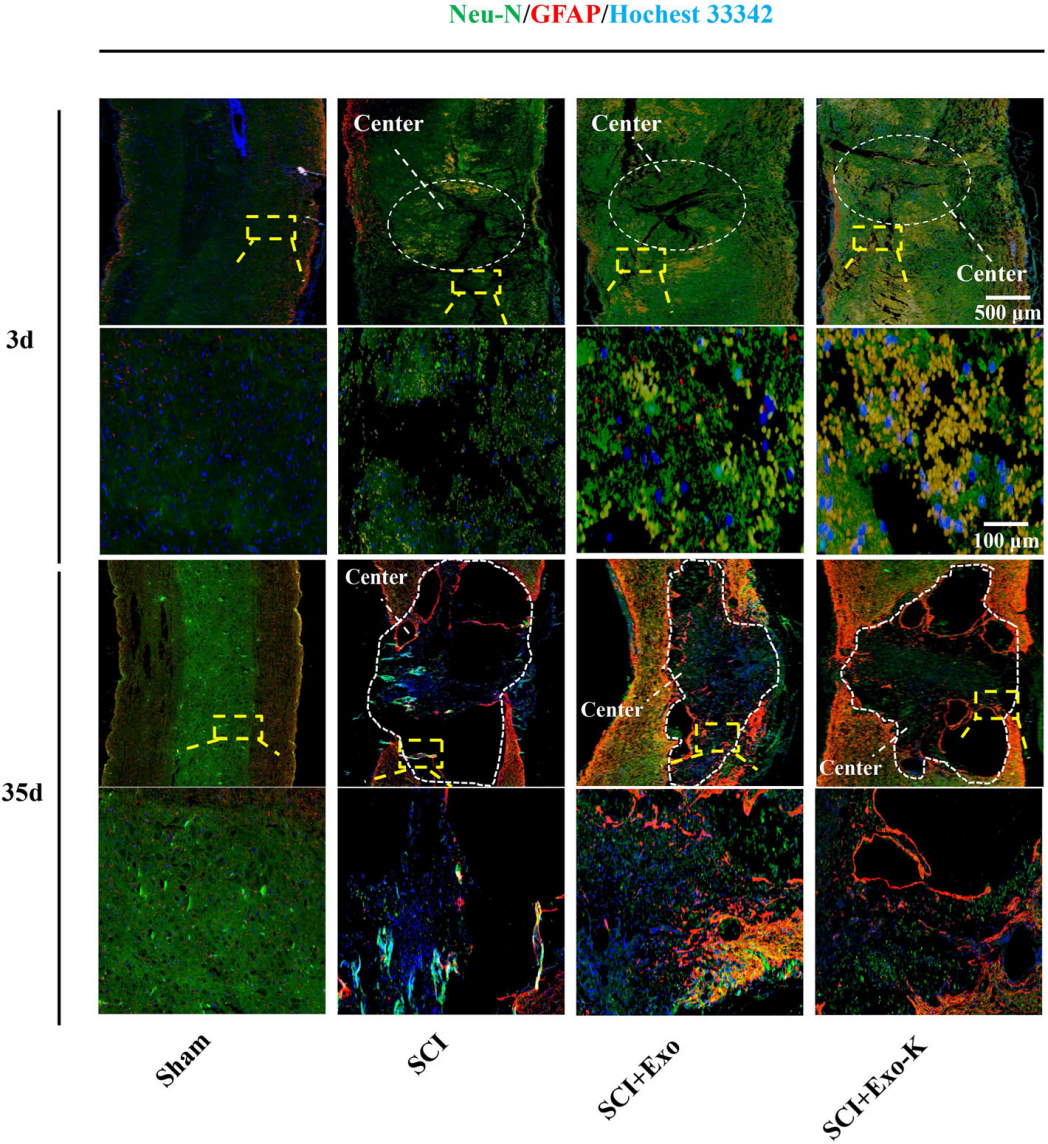
Preserved neurons in four groups of rats identified by Neu-N. Representative immunofluorescence images of Neu-N/GFAP Exo-K in sham-operated rats, SCI rats, SCI rats treated with Exo, and SCI rats treated with Exo-K at 3 DPI and 35 DPI. Scale bar = 500 μm, Scale bar = 100 μm

Furthermore, we pretreated PC12 cells, which were subsequently stimulated by NGF and LPS, with MSC-Exo to investigate whether exosomal miR-199a-3p/145-5p were indispensable in NGF-induced PC12 differentiation. Our results showed that neuronal marker expression (NF-H, β-tubulin-III, Neu-N) suppressed by LPS could be upregulated by Exo; however, this protective effect was partially abolished by knockdown of miR-199a-3p/145-5p in MSC-Exo (Fig. 2g–i). The results also showed that the downregulation of p-Akt and p-Erk induced by LPS was also reversed by increasing doses of MSC-Exo (Fig. 2k, l). Moreover, the inhibition of PC12 neurite outgrowth induced by LPS, which was partially reversed by MSC-Exo, was closely related to exosomal miR-199a-3p/145-5p expression (Fig. 2m, n). More importantly, we observed that neurons were greatly preserved at 3- and 35-days post injury (DPI) as demonstrated by Neu-N staining (Fig. 3). The compromised effect of Exo-K on the promotion of neurite outgrowth, which was inhibited by LPS, was also observed in primary neurons in vitro (see Additional file 9A). Thus, the presence of miR-199a-3p/145-5p recapitulated the neuroprotective effects of MSC-Exo, while miR-199a-3p/145-5p knockdown suppressed these effects. Moreover, we found that miR-199a-3p/145-5p levels in MSC-Exo were correlated with increased endothelial cell migration and tube formation abilities, which were suppressed by LPS (see Additional file 9B-E), while these effects were compromised by MSC-Exo-K. This finding may explain why exosomes are often administered in the early stage of SCI, as the blood supply is potentially impacted as well. In summary, miR-199a-3p/145-5p participated in MSC-Exo-mediated neurite outgrowth in vitro and in vivo.

### miR-199a-3p/145-5p promoted PC12 cell differentiation by regulating downstream factors of NGF/TrkA

**Fig. 4:**
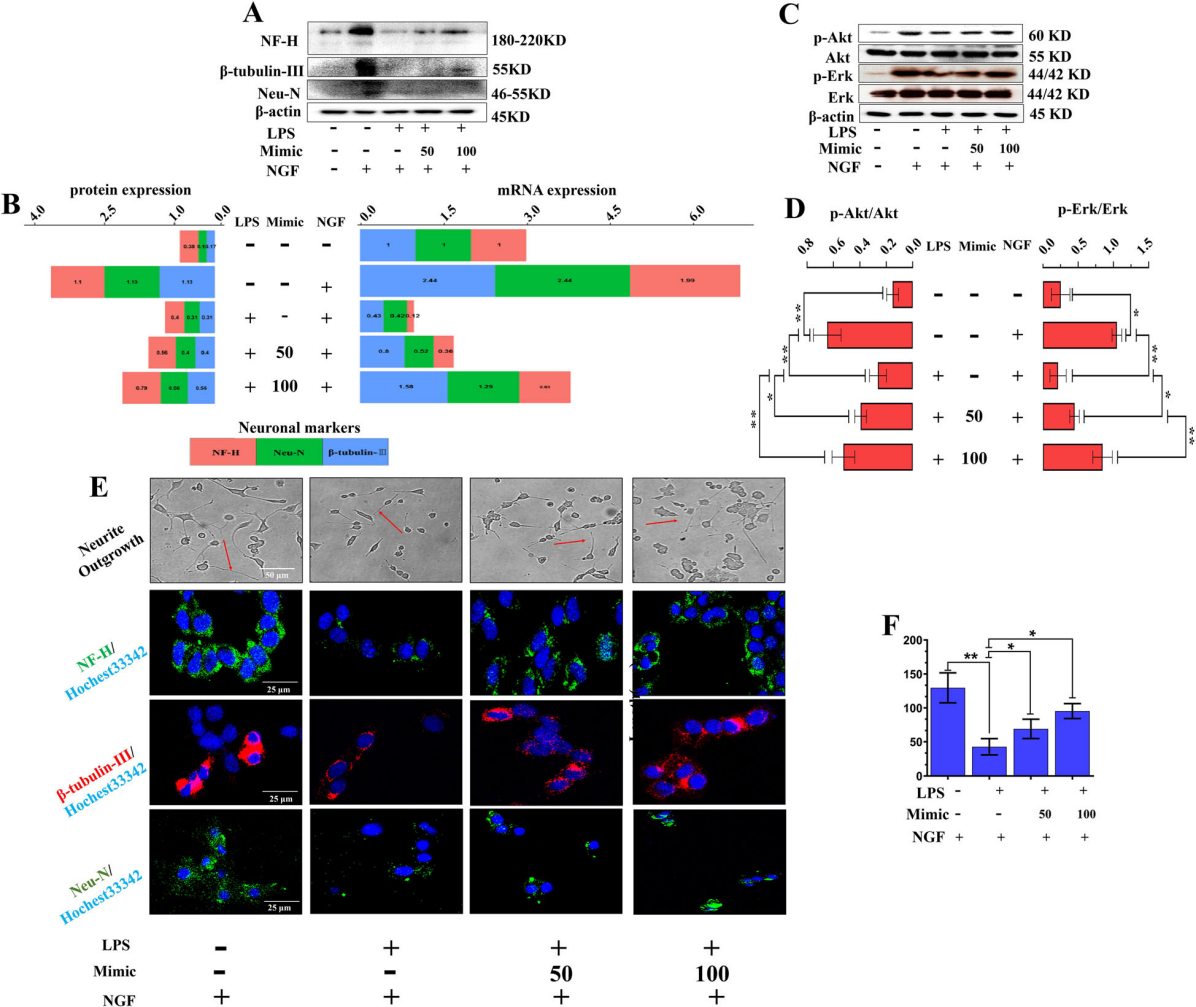
miR-199a-3p promoted the LPS-induced inhibition of PC12 cell differentiation by regulating downstream factors of NGF/TrkA. **a**, **b** Representative western blot images and quantitative analysis of NF-H, β-tubulin-III, and Neu-N gene expressions in LPS- and NGF-stimulated PC12 cells pre-transfected with different doses of miR-199a-3p mimics. Data are represented as average value of 3 independent experiments. **c**, **d** Representative western blot images and quantitative analysis of western blots of p-Akt and p-Erk expression (*n* = 3). Data are represented as mean ± SD. **e**, **f** Representative immunofluorescence images and quantitative analysis of cell neurite growth (red arrow) and neuronal marker expression (NF-H, β-tubulin-III, and Neu-N) in LPS- and NGF-stimulated PC12 cells pretreated with different doses of miR-199a-3p mimics (*n* = 3). Data are represented as mean ± SD. Scale bar = 50 μm. *p < 0.05; **p < 0.01, LPS lipopolysaccharide, NGF neuronal growth factor

**Fig. 5:**
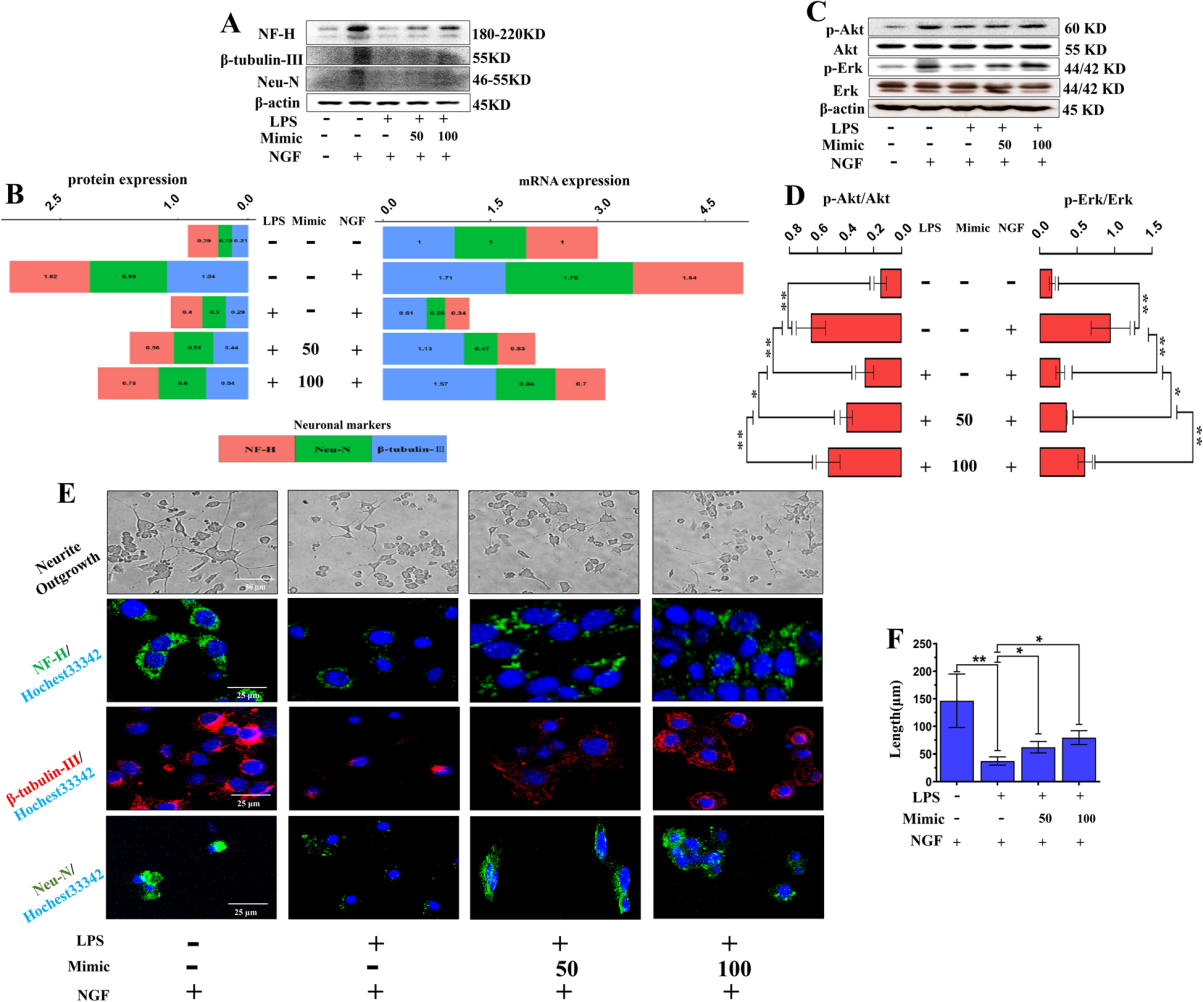
miR-145-5p promoted LPS-induced inhibition of PC12 cell differentiation by regulating downstream factors of NGF/TrkA. **a**, **b** Representative images of western blots and quantitative analysis of NF-H, β-tubulin-III, and Neu-N gene expressions in LPS- and NGF-stimulated PC12 cells pre-transfected with different doses of miR-145a-5p mimics. Data are represented as average value of 3 independent experiments. **c**, **d** Representative western blot images and quantitative analysis of p-Akt (I) and p-Erk (J) expression in LPS- and NGF-stimulated PC12 cells pre-transfected with different doses of miR-145-5p mimics (*n* = 3). Data are represented as mean ± SD. **e**, **f** Representative immunofluorescence images and quantitative analysis of cell neurite growth (red arrow) and neuronal marker expression (NF-H, β-tubulin-III, and Neu-N) in LPS- and NGF-stimulated PC12 cells pretreated with different doses of miR-145a-5p mimics (*n* = 3). Data are represented as mean ± SD. Scale bar = 50 μm. *p < 0.05; **p < 0.01. LPS lipopolysaccharide, NGF neuronal growth factor

To investigate whether exogenous miR-199a-3p/145-5p showed similar positive effects on neurite outgrowth, we pretransfected miR-199a-3p/145-5p mimics into PC12 cells before treatment with LPS or NGF. The results revealed that LPS dramatically reduced neuronal marker (NF-H, β-tubulin-III, Neu-N) expression at the protein and mRNA levels, which was rescued by miR-199a-3p/145-5p mimics in a dose-dependent manner (Fig. 4a, b; Fig. 5a, b). The significant suppression of p-Akt and p-Erk by LPS, which could be highly activated by NGF, was also partially promoted by miR-199a-3p/145-5p mimics in a dose-dependent manner (Fig. 4c, d; Fig. 5c, d). Furthermore, increasing doses of miR-199a-3p/145-5p mimics also led to dose-dependent promotion of LPS-stimulated PC12 neurite outgrowth, and neuronal markers (NF-H, β-tubulin-III, Neu-N) were examined and visualized by confocal microscopy (Fig. 4e, f; Fig. 5e, f). This observation suggests that miR-199a-3p/145-5p are involved in the NGF/TrkA pathway and PC12 cell differentiation.

### miR-199a-3p and miR-145-5p targeted the Cblb and Cbl genes, respectively

**Fig. 6:**
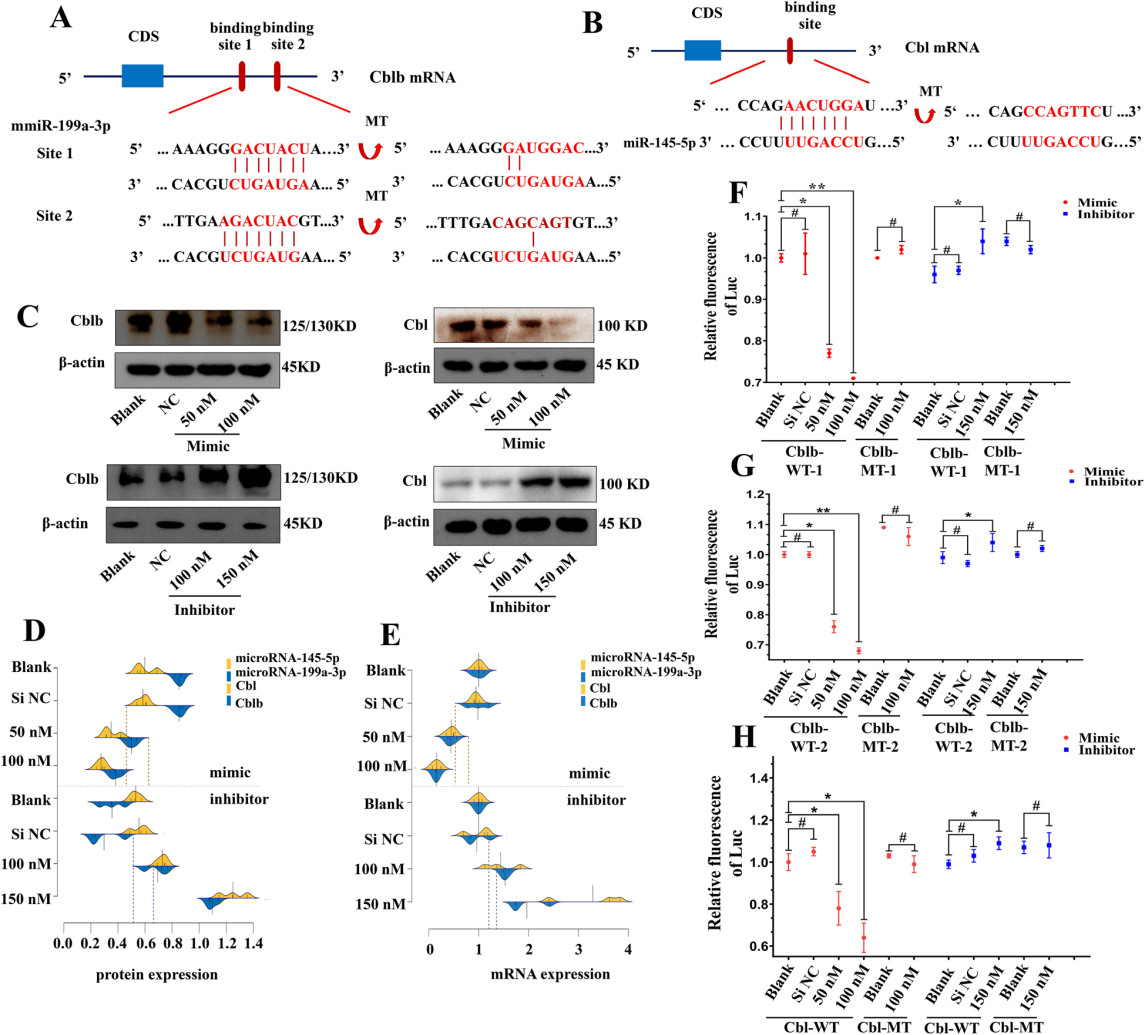
miR-199a-3p and miR-145-5p targeted the Cblb and Cbl genes, respectively. Predicted binding sites of miR-199a-3p (**a**) and miR-145-5p (**b**) on Cblb and Cbl mRNAs. **c**–**e** Cblb and Cbl gene expressions in PC12 cells transfected with different doses of miR-199a-3p/145-5p mimics and inhibitors assayed by western blot and QRT-PCR (*n* = 3). Data are represented as beanplot exhibiting values from 3 independent experiments. Luciferase reporter assay of the first predicted binding site (**f**) and the second predicted site (**g**) of miR-199a-3p in HEK293 cells. Data are represented as mean ± SD. **h** Luciferase reporter assay of the predicted binding site of miR-145-5p in HEK293 cells (*n* = 3). Data are represented as mean ± SD. *p < 0.05; **p < 0.01; #*p* > 0.05. WT wild type, MT mutant, QRT-PCR quantitative real-time polymerase chain reaction

Through bioinformatics prediction, we hypothesized that Cblb and Cbl were direct targets of miR-199a-3p and miR-145-5p, respectively (Fig. 6a, b). To investigate whether miR-199a-3p regulated Cblb gene expression at the transcriptional or posttranscriptional level, we transfected both miR-199a-3p/145-5p mimics and inhibitors into PC12 cells. Western blotting and QRT-PCR showed that Cblb, and Cbl protein and mRNA expressions decreased with increasing doses of the mimics and increased with increasing doses of the inhibitors (Fig. 6c–e). These results suggested that miR-199a-3p/145-5p suppressed Cblb and Cbl expression in PC12 cells at the transcriptional and posttranscriptional levels.

Furthermore, we cloned the two predicted wild-type binding sites (WT-1/-2) and the mutant sites into plasmids and cotransfected the pGL3-reporter and pRL-TK vector with miRNA mimics or inhibitors into HEK293 cells. A previous study predicted a relationship between miR-199a-3p and Cblb, while our results demonstrated a direct interaction between them. Significant changes in relative luciferase activity in HEK293 cells transfected with scramble sequence (Si NC group) were not observed, while decreased luciferase activity along with increasing doses of the miR-199a-3p mimic was determined (Fig. 6f, g). Increased luciferase activity in HEK293 cells was also observed when cells were transfected with 150 nm miR-199a-3p mimics (Fig. 6f, g). Regardless of whether the cells were transfected with miR-199a-3p mimics or inhibitors, the luciferase activity of HEK293 cells transfected with a vector carrying mutant sites was not affected (Fig. 6f, g).

Regardless of whether the cells were transfected with miR-145-3p mimics or inhibitors, the luciferase activity of HEK293 cells transfected with a vector carrying the mutant site was not significantly changed (Fig. 6h). The scramble control did not influence luciferase activity in HEK293 cells. These results suggested that miR-199a-3p/145-5p suppressed Cblb and Cbl expression via direct binding to their predicted mRNA seed regions.

### Cblb and Cbl regulated the NGF/TrkA pathway through modulation of TrkA ubiquitination

**Fig. 7:**
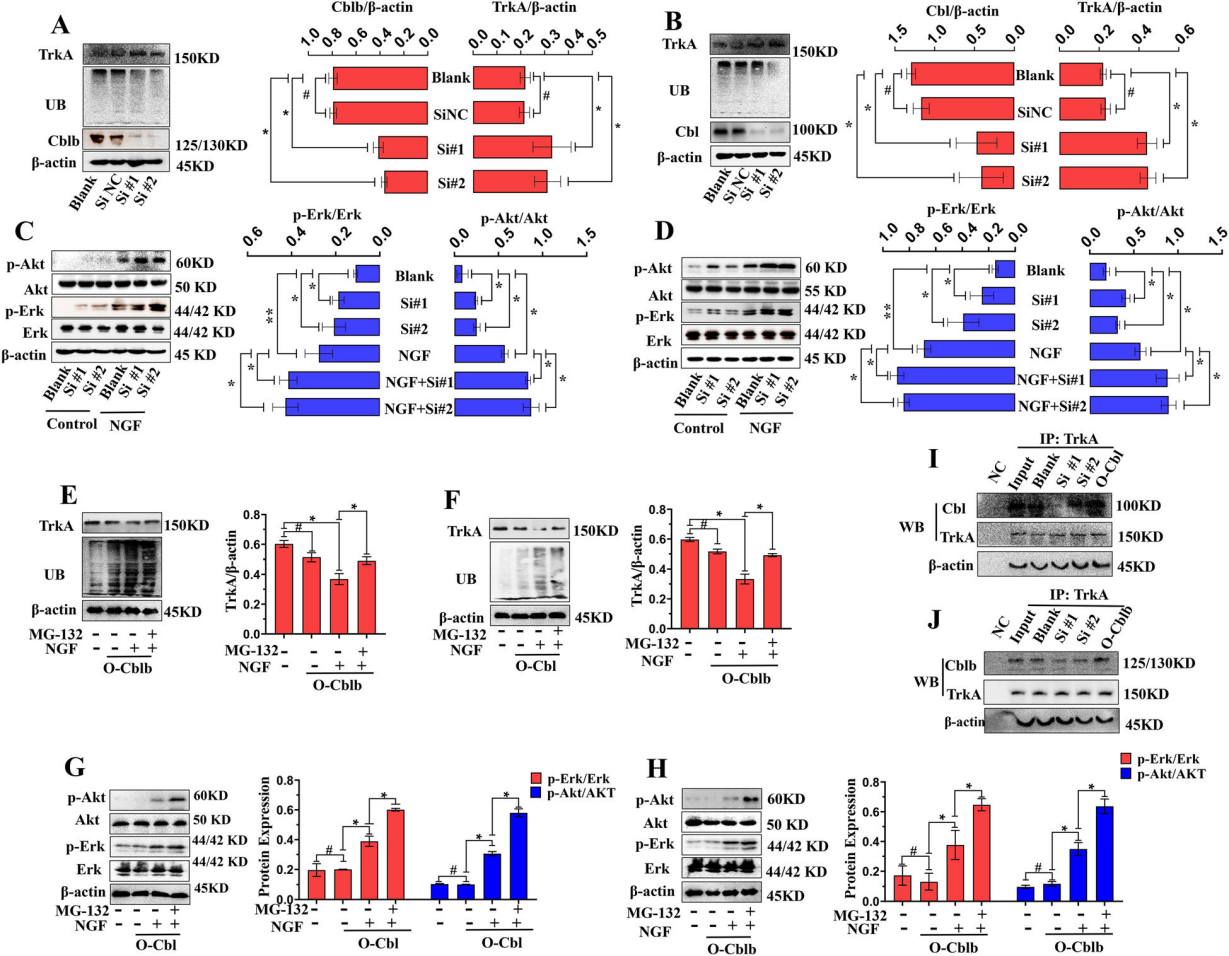
Cblb and Cbl regulated the NGF/TrkA pathway through modulation of TrkA ubiquitination. Representative images and quantitative analysis of total ubiquitinated proteins and TrkA in nontransfected PC12 cells and Cblb (**a**) (*n* = 3) and Cbl (**b**) (*n* = 3) knockdown PC12 cells. Data are represented as mean ± SD. Representative images and quantitative analysis of p-Erk and p-Akt in nontreated PC12 cells and Cblb (**c**) and Cbl (**d**) knockdown PC12 cells. Data are represented as mean ± SD. Representative images and quantitative analysis of total ubiquitinated proteins and TrkA in nontreated PC12 cells and Cblb (**e**)- and Cbl (**f**)-overexpressing PC12 cells stimulated with NGF and MG-132 (*n* = 3). Data are represented as mean ± SD. **i** Representative images and quantitative analysis of p-Erk and p-Akt in nontreated PC12 cells and Cblb (**g**)- and Cbl (**h**)-overexpressing PC12 cells stimulated with NGF and MG-132 (n = 3). Data are represented as mean ± SD. Representative coimmunoprecipitation images of TrkA and Cblb (**i**) and TrkA and Cbl (**j**) in PC12 cells. *p < 0.05; **p < 0.01; #*p* > 0.05

To further explore how Cblb and Cbl interact with the NGF/TrkA pathway, we knocked down or overexpressed these two genes in PC12 cells. First, we designed three siRNAs against the Cblb and Cbl genes, two of which (Si #1 and Si #2) were more effective in suppressing Cblb and Cbl mRNA expression (see Additional file 10A-B; 10D-E). When Cblb and Cbl were knocked down by the siRNAs, total ubiquitinated proteins in PC12 cells were greatly reduced; however, a marked increase in TrkA was observed compared with that in nontreated cells (Fig. 7a, b). Single knockdown of Cblb or Cbl caused upregulation of p-Erk and p-Akt, and NGF activated p-Erk and p-Akt (Fig. 7c, d), subsequently promoting NGF-induced neurite outgrowth in PC12 cells (see Additional file 10C; 10F).

Then, we successfully upregulated Cblb and Cbl expression in PC12 cells (see Additional file 11). Overexpression of Cblb and Cbl led to higher ubiquitination levels of total proteins and lower expression of TrkA (Fig. 7e, f). NGF stimulation accelerated the degradation process of TrkA when Cblb and Cbl were overexpressed, but this process was inhibited by the proteasome inhibitor MG-132 (Fig. 7e, f). We also found that NGF-activated p-Erk and p-Akt were further upregulated in Cblb- and Cbl-overexpressing cells by the protease inhibitor MG-132 (Fig. 7g, h). More importantly, we pulled down Cblb and Cbl proteins in PC12 cells with knockdown and overexpression of Cblb and Cbl by using a primary antibody against TrkA, further confirming the direct interaction between Cblb and TrkA (Fig. 7i, j).

### Exosomal miR-199a-3p/145-5p alleviated damage to the lesion site and facilitated locomotor function in SCI rats

**Fig. 8:**
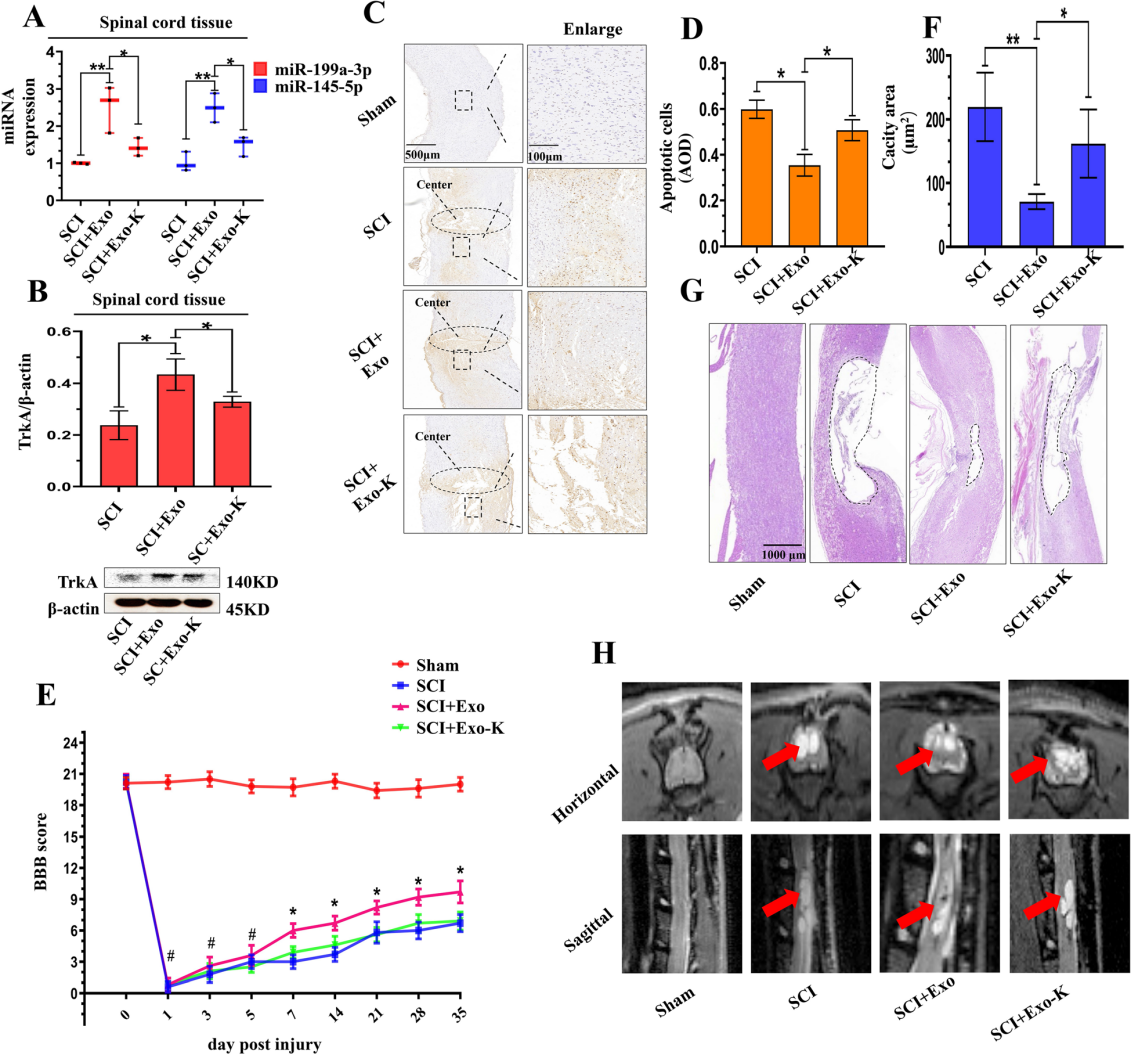
Function of exosomal miR-199a-3p/145-5p in vivo in SCI rats. **a** Relative miR-199a-3p/145-5p expression levels in SCI and SCI of MSC-Exo- and Exo-K-treated rats at 3 DPI (*n* = 3). Data are represented as mean ± SD. **b** Representative images and quantitative analysis of TrkA in SCI, SCI treated with Exo, and SCI treated with Exo-K rats at 3 DPI (*n* = 3). Data are represented as mean ± SD. **c**, **d** Representative images and quantitative analysis of TUNEL staining in sham-operated rats, SCI rats, SCI rats treated with Exo, and SCI rats treated with Exo-K 3 DPI (*n* = 3). Scale bar = 500 μm, scale bar = 100 μm. Data are represented as mean ± SD. **e** BBB scores of the four groups of rats (sham, SCI, SCI + Exo, and SCI + Exo-K) on the 1st, 3rd, 5th, 7th, 14th, 21st, 28th, and 35th DPI (*n* = 10/group). Data are represented as mean ± SD. **f**, **g** Quantitative analysis and representative images of the lesion size (HE staining) in the four groups of rats at 35 DPI (*n* = 10). Data are represented as mean ± SD. Scale bar = 1000 μm. **h** Representative MRI images of the lesion area (red arrow) of rats at 35 DPI. *p < 0.05; #*p* > 0.05. SCI, spinal cord injury; DPI, day post injury; MSC-Exo, MSC-derived exosomes; Exo-K, MSC-Exo with miRNA inhibition; BBB, Basso, Beattie and Bresnahan; HE, hematoxylin-eosin

In vivo, we injected the rats with PBS, Exo, and Exo-K via the tail vein. The high expression of miR-199a-3p and miR-145-5p in rats receiving Exo injection was partially downregulated in rats receiving Exo-K injection, which implied the successful delivery of the miRNAs by exosomes (Fig. 8a). Furtherly, track of PKH26-labeled exosomes by immunofluorescence in vivo proved this point (see Additional file 12). We also observed that the increase in TrkA in rats receiving Exo injection was partly downregulated in rats receiving Exo-K injection at 3 DPI (Fig. 8b). Exo injection exerted an antiapoptotic effect on the lesion site, while this effect was abrogated with the inhibition of miR-199a-3p/145-5p (Fig. 8c, d). Decreased inflammatory levels in vivo were also partially attributed to exosomal miR-199a-3p/145-5p levels (see Additional file 13).

**Fig. 9:**
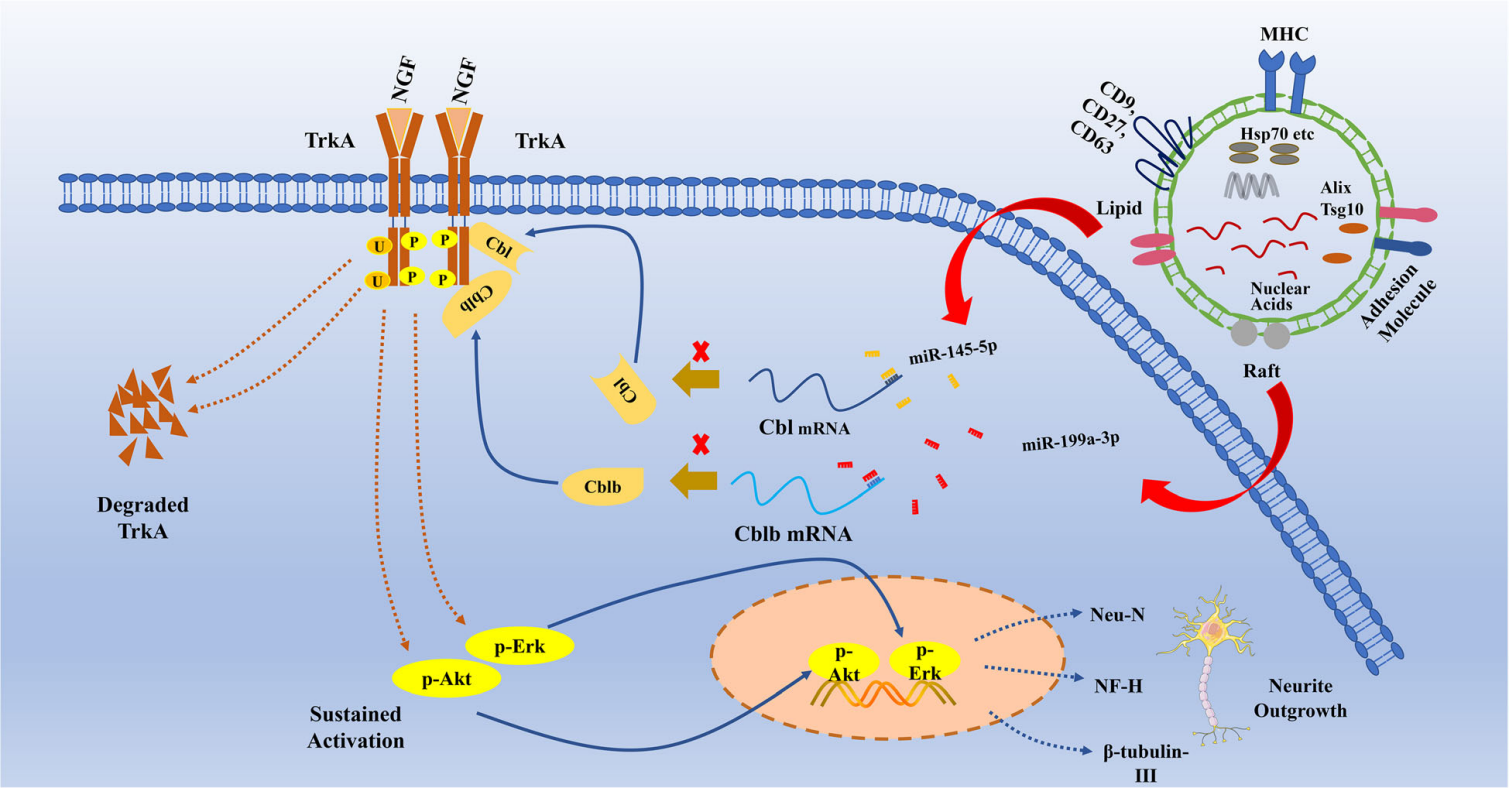
Signaling pathways affected by exosomal miR-199a-3p/145-5p. Exogenous exosomes from human umbilical cord mesenchymal stem cells are taken up by damaged neurons and release abundant levels of miRNA-199a-3p/145-5p, which target Cblb and Cbl mRNAs to inhibit the ubiquitination-mediated degradation of TrkA. Sustained activation of p-Erk and p-Akt leads to persistent expression of Neu-N, NF-H, β-tubulin-III, and neurite outgrowth

The Basso, Beattie and Bresnahan (BBB) scale was used to assess the locomotor function of the hindlimbs of rats, and the values were recorded from onset to 35 DPI. Strongly impaired locomotor function of the hindlimbs of rats was observed on the 1st DPI, and significant improvement was detected on the 7th DPI (Fig. 8e). Hematoxylin-eosin (HE) staining showed that the damage at the lesion site of SCI rats was smaller than that of SCI + Exo rats. Interestingly, this protective effect was partially suppressed when miR-199a-3p/145-5p were inhibited in MSC-Exo (Fig. 8f, g). We further detected spinal cord damage in vivo, and the MRI results indicated that MSC-Exo greatly decreased the lesion size of the injured spinal cord, while Exo-K exerted weaker protective effects on the damaged spinal cord (Fig. 8h). These data indicated the pivotal roles of miR-199a-3p/145-5p in MSC-Exo-mediated treatment of SCI in vivo (Fig. 9). Cbl and Cbl inhibition by exosomal miR-199a-3p/145-5p emphasized the neuroprotective effect of MSC-Exo.

## Discussion

miRNAs, noncoding RNAs of approximately 22 nucleotides, depending on the exosomal origins, are markedly enriched in exosomes and target mRNAs by binding to their 3′UTRs [16, 17]. The hUC-MSC-derived exosomal miRNA expression profiles first identified by Fang using QRT-PCR (miR-21, -23a, -125b, -145) [18] and then quantified by us using absolute quantitative sequencing shared similar results. Cargo miRNAs, as the most abundant nucleotides in exosomes, are receiving increasing attention from researchers, mainly owing to the improved understanding of miRNAs and advances in the techniques used to investigate miRNAs.

Findings showing attenuated central inflammation in rats through detection of IL-1, IL-6, and TNF-α; upregulation of proinflammatory cytokines (IL-4, IL-10); and reduction in apoptotic neurons in the early process of exosomal treatment [14] were consistent with our results. Improvement of the microenvironment at the lesion site enhanced neurite outgrowth-related p-Akt/p-Erk pathway activation by NGF in PC12 cells and decreased secondary apoptosis of neurons. However, there is less evidence that exosomal miRNAs are involved in the protection of neurons at the early stage of SCI, and the underlying mechanism remains unclear. The injury model of PC12 cells was established by using LPS instead of other inflammatory cytokines as previously reported [19] because both LPS model and other inflammatory models truly reflect the damaged status of neurons under inflammation [20, 21]. Through knockdown of mir-199a-3p/145-5p in exoxomes, we demonstrated the crucial role of mir-199a-3p/145-5p in the neuronal differentiation promoted by exosomes. Furtherly, we predicted that Cblb and Cbl genes are targeted by miR-199a-3p and miR-145-5p.

The protooncogenes Cbl and Cblb have been suggested to have a negative effect on RTKs, and the regulation of both genes is analogous to that of T cell receptor and epidermal growth factor (EGFR) [22–25]. As demonstrated for RTKs, E3 ubiquitin ligases such as NEDD4-2, tumor necrosis factor receptor-associated factor 6 (TRAF6), Cbl, and Cblb facilitate TrkA ubiquitination, internalization, and turnover [26–28]. Ubiquitins (Cbl and Cblb) are conjugated onto TrkA along with the onset of self-phosphorylation of intracellular peptide fragments of TrkA, which can be suppressed by exosomal miR-199a-3p/145-5p in vivo and in vitro. Through an in vitro study, we determined that the Cbl mRNA 3′UTR was the target of miR-145-5p and that the Cblb mRNA 3′UTR was the target of miR-199a-3p. The predicted biding site of Cbl mRNA for miR-145-5p is first reported and proved by us; interestingly, the predicted biding site of Cblb mRNA for miR-199a-3p was previously reported but not examined in RAW264.7 cells by Rong [29].

In the absence of the Cbl and Cblb genes, an activation of the p-Akt/p-Erk pathways and increased neurite growth stimulated by NGF were observed. However, both the p-Akt/p-Erk pathways were soon downregulated, which was associated with rapid degradation of TrkA in response to NGF stimulation and the overexpression of Cblb and Cbl genes. Similar to Takahashi’s results [27–30], our co-IP data showed that TrkA ubiquitination and degradation were statistically affected by Cbl, but the domain of TrkA required for ubiquitination by Cbl remains unclear. The mechanisms of the TrkA-Cblb interaction may involve the TKB domain (an SH2-like domain) promoting the binding of phosphorylated tyrosine on activated TrkA and Src homology 2 adapter protein 2 (SH2B2, also known as APS) [31]. We showed from two aspects that the levels of Cblb and Cbl proteins, which could be clearly reduced by exosomal miRNAs, were associated with neurite outgrowth in PC12 cells and were potential influencing factors for SCI by affecting the turnover and/or activation of TrkA and maintaining sustained activation of p-Erk/p-Akt and neurite outgrowth. Intriguingly, this theory is potentially suitable for other E3 ubiquitin ligases, other Trk members regulating neurite outgrowth, and these E3 ubiquitin ligases are underlying targets of exosomes that are enriched for regulatory noncoding RNAs. Furthermore, knockdown of both miRNAs in exosomes directly led to lower expression of TrkA in vivo and impaired neurological function in SCI rats, which emphasized the effects of miR-199a-3p/145-5p in vivo.

Even though multiple drugs, such as nonsteroidal anti-inflammatory drugs (NSAIDs), anti-CD11d antibodies, minocycline, progesterone, estrogen, riluzole, polyethylene glycol (PEG), astatin and inosine, are already used in human clinical applications, satisfying outcomes are rarely obtained [32]. The use of exosomes in treating SCI is mainly due to the therapeutic effect of stem cells, which is thought to be a result of paracrine mechanisms, and an increasing number of researchers speculate that exosomes are a promising next-generation cell-free agent. However, rigorous assessment of their efficacy and safety and detailed mechanisms of action are urgently needed before or in conjunction with clinical application.

## Conclusions

In summary, miR-199a-3p/145-5p derived from hUC-MSCs could promote locomotor functional recovery of SCI rats potentially by targeting the Cblb and Cbl genes, which resulted in modulation of the turnover and/or activation of TrkA. Furthermore, exosomes tended to promoted neurite outgrowth by decreasing the inflammatory level at the lesion site of the spinal cord, creating a favorable environment for neurite outgrowth.

## Supplementary Information

**Additional file 1.** Animal protocol.

**Additional file 2.** Supplementary methods and materials.

**Additional file 3.** SiRNA and miRNA sequences.

**Additional file 4.** Coding Sequence of Cbl and Cblb Genes.

**Additional file 5.** Primers of target genes.

**Additional file 6.** Outcome of miRNA sequencing.

**Additional file 7.** Bioinformatic analysis of miRNAs. (A) The linear relationship between lgSD-2 and lgTPM. (B) Predicted target genes of the top 20 miRNAs (plus miR-145-5p) in miRanda. TPM, transcripts per million; SD, standard difference.

**Additional file 8.** Images of MSC-Exo taken up by PC12 cells and miR-199a-3p and miR-145-5p expression profiles in different cells. (A) Representative images of MSC-Exo taken up by PC12 cells. Scale bar=25 μm. (B) The morphology of primary neurons, astrocytes, endothelial cells and meningeal fibroblasts visualized by an inverted fluorescence microscope. Scale bar=50 μm. MSC-Exo, MSC-derived exosomes; QRT-PCR, quantitative real-time polymerase chain reaction.

**Additional file 9.** The effects of exosomal miR-199a-3p/miR-145-5p on endothelial cell migration, tube formation, and neurite outgrowth of primary neurons in vitro. (A) Representative images of neurite outgrowth (red arrow) in LPS-stimulated primary neurons pretreated with Exo and Exo-K. Scale bar=50 μm. (B) Representative images of cell migration in LPS-stimulated endothelial cells pretreated with Exo and Exo-K (*n*=3). Scale bar=50 μm. (C) Representative images of tube formation in LPS-stimulated endothelial cells pretreated with Exo and Exo-K (n=3). Scale bar=50 μm. (D) Quantitative analysis of migrated cells in (B) and (E) the length of the tubes in (C). Data are represented as mean ± SD. LPS, lipopolysaccharide; MSC, mesenchymal stem cell; MSC-Exo, MSC-derived exosomes; Exo-K, MSC-Exo with the inhibition of miRNAs.

**Additional file 10.** Knockdown of Cblb and Cbl increased NGF-induced neurite outgrowth in PC12 cells. Cblb mRNA level in nontransfected PC12 cells and PC12 cells transfected with scramble sequence, Cblb SiRNA #1, SiRNA #2, and SiRNA #3 as detected by QRT-PCR (A). Data are represented as mean ± SD. Quantitative analysis (B) and representative images (C) of cell neurite growth in NGF-stimulated PC12 cells transfected with scramble sequence, Cblb SiRNA #1 and SiRNA #2. Scale bar=50 μm. Data are represented as mean ± SD. Cbl mRNA level in nontransfected PC12 cells and PC12 cells transfected with scramble sequence, Cbl SiRNA #1, SiRNA #2, and SiRNA #3 as detected by QRT-PCR (D). Data are represented as mean ± SD. Quantitative analysis (E) and representative images (F) of cell neurite growth in NGF-stimulated PC12 cells transfected with scramble sequence, Cblb SiRNA #1 and SiRNA #2. Scale bar=50 μm. Data are represented as mean ± SD. NGF, neuronal growth factor; QRT-PCR, quantitative real-time polymerase chain reaction.

**Additional file 11.** Overexpression of Cblb and Cbl in PC12 cells. (A) Quantitative analysis of Cblb mRNA expression by QRT-PCR (B) and representative western blot images of Cblb. (C) Quantitative analysis of Cbl mRNA expression by QRT-PCR (B) and representative western blot images of Cbl. Data are represented as mean ± SD. QRT-PCR, quantitative real-time polymerase chain reaction.; EGFP; enhanced green fluorescent protein.

**Additional file 12.** Track of PKH26-labelled exosomes in vivo by confocal microscope. The nucleus (blue), Neu-N (green) and PHK26 (red and indicated by the red arrow) were stained to identify the uptake of exosomes by the injured neurons.

**Additional file 13.** Exosomal 199a-3p/145-5p strongly reduced inflammation levels. Representative western blot images (A) and quantitative analysis of IL-1 (B), IL-6 (C) and TNF-α (D). Data are represented as mean ± SD.

## Data Availability

Not applicable.

## Abbreviations

SCI: Spinal cord injury
LPS: Lipopolysaccharide
hUC-MSC: Human umbilical cord mesenchymal stem cell
EV: Extracellular vesicles
NGF: Neuronal growth factor
SNT: Suc-associated neurotrophic factor-induced tyrosine-phosphorylated target
FGF: Fibroblast growth factor
EGF: Epidermal growth factor
PDGF: Platelet-derived growth factor
MSC-Exo: MSC exosomes
SD: Standard difference
TPM: Transcripts per million
Exo-K: Exosomes with the inhibition of miR199a-3p/145-5p
PC12 cell: (rat) Pheochromocytoma cell
RT-QPCR: Quantitative real-time polymerase chain reaction
RARβ: Retinoic acid receptor β
MAG: Myelin-associated glycoprotein
PLP: Proteolipid protein
PTEN1: Phosphatase and tensin homolog pseudogene
circRNAs: Circular ribonucleic acids
IFN: Interferon
G-CSF: Granulocyte-colony stimulating factor
MCP-1α: Monocyte chemotactic protein 1α
RTK: Receptor tyrosine kinase
MVBs: Multi-vesicular bodies
EGFR: Epidermal growth factor receptor
TRAF6: Tumor necrosis factor receptor-associated factor 6
SH2B2: src homology 2 adapter protein 2
NSAIDs: Non-steroidal anti-inflammatory drugs
PEG: Polyethylene glycol
