# Supplementary material for: Umbilical mesenchymal stem cell-derived exosomes facilitate spinal cord functional recovery through the miR-199a-3p/145-5p-mediated NGF/TrkA signaling pathway in rats
Source: Stem Cell Res Ther. 2021 Feb 12;12:117. doi: 10.1186/s13287-021-02148-5 (PMC7879635; doi:10.1186/s13287-021-02148-5)
Supplement: Supplementary file 1 — Additional file 1. Animal protocol. [file 13287_2021_2148_MOESM1_ESM.docx]

| **Group**  **Day** | Sham (n=18) | SCI (n=19) | SCI+Exo (n=20) | SCI+Exo-K (n=19) |
| --- | --- | --- | --- | --- |
| 0 | primary cells, n=5 | NA | NA | NA |
| 1 | NA | NA | exosome tracking, n=1 | NA |
| 3 | IF, Tunnel staining,  n=3 | IF, Tunnel staining,  n=3 | IF, Tunnel staining,  n=3 | IF, Tunnel staining,  n=3 |
| 3 | NA | WB, n=3; QPCR,  n=3 | WB, n=3; QPCR,  n=3 | WB, n=3;  QPCR,  n=3 |
| 35 | BBB score, IF, HE,  n=10 | BBB score, IF, HE,  n=10 | BBB score, IF, HE,  n=10 | BBB score, IF, HE,  n=10 |

**Additional file 1. Animal protocol**

SCI, spinal cord injury; Exo, exosomes; Exo-K, exosomes with the inhibition of miR-199a-3p/145-5p; IF, immunofluorescence; BBB, Basso,Beatlie, Bresnahan; HE, hematoxylin and eosin; NA, not available.
