# Supplementary material for: Umbilical mesenchymal stem cell-derived exosomes facilitate spinal cord functional recovery through the miR-199a-3p/145-5p-mediated NGF/TrkA signaling pathway in rats
Source: Stem Cell Res Ther. 2021 Feb 12;12:117. doi: 10.1186/s13287-021-02148-5 (PMC7879635; doi:10.1186/s13287-021-02148-5)
Supplement: Supplementary file 2 — Additional file 2. Supplementary methods and materials. [file 13287_2021_2148_MOESM2_ESM.docx]

**Additional file 2: Supplementary methods and materials**

**MSC characterization**

hUC-MSCs, within passage five, were purchased from Guangzhou selera Stem Cell Technology Co., Ltd and characterized by surface markers using flow cytometry. Primary antibodies conjugated with fluorescein, including antibodies against CD73 (Abcam, UK), CD90 (Abcam, UK), CD105 (Abcam, UK), CD34 (Abcam, UK), CD45 (Abcam, UK) and HLA-DR (Abcam, UK), were co-incubated with stem cells. Subsequently, flow cytometry (BD Biosciences, Franklin Lakes, New Jersey, USA) was used to analyze fluorescence intensity of each group. Data were exported using FlowJo software 10.0 (Stanford University, Palo Alto, USA).

**Exosomes labeling**

hUC-MSCs derived exosomes were labeled with PKH26 dye (Sigma-Aldrich, St. Louis, MO, USA) or following the manufactures’ protocols. Then, the pelleted exosomes were suspended in 10 mL PBS and re-ultracentrifuged (4°C, 100, 000 g for 60 min). The sediments were re-suspended in sterile PBS and stored at 4℃ up to a week.

**Cell proliferation assay**

The proliferation of PC12 cells was detected using the Cell Counting Kit-8 (CCK-8; Dojindo, Japan) following the manufacturer’s instructions. The absorbance at 450 nm was measured using a microplate reader (Biotek, Winooski, VT, USA). Optical density (OD) was converted to cell viability via the formula: $cell viability=\frac{/Experiment\left( \mathrm{OD} \right)-Blank(OD)/}{\mathrm{Control}\left( \mathrm{OD} \right)-Blank\left( \mathrm{OD} \right)}$.

**miRNA Sequencing**

Sequencing analysis of miRNAs from MSC-Exo was performed by Illumia platform by KangCe Biotechnology Co., Ltd in WuHan (Wuhan, China). The miRNA profiles were quantified by transcripts per million (TPM). Exosomes derived from hUC-MSC were immediately frozen in liquid nitrogen for use. Total RAN was purified and amplified following the instructions of Seramir Exosome RNA Amplification kit (SBI, California, USA).

**Cells isolation**

***Neurons***

Neurons and artery endothelial cells were isolated as previously describe^[1-5]^. The spinal cord tissue was mechanically minced and digested (0.25% tyrosine). After centrifugation (1000 g for 5 min), cells were re-suspended in corresponding medium with or without 10% fetal bovine serum (FBS; Gibco, Carlsbad, USA).

***Endothelial cells***

When spine cord was immediately removed from the rat, a second researcher cut open the abdomen and swiftly isolated the aorta (about 1.5 cm long). Subsequently, we minced the aorta into vascular rings and digested these rings in 0.25% tyrisin. Then, we centrifuged the cell suspension, and re-suspended the debris with endothelial cell growth medium-2 (EGM-2; Lonza, Basel, Switzerland) with 10% FBS. The endothelial cells were cultured in a 75cm^2^ culture flask with a replacement of the culture every other two days. Four type of cells were characterized by immunofluorescence (IF).

**Cell migration**

The tube formation and cell migration assays were performed as previously depicted^[6]^. Endothelial cells were pretreated with LPS (Sigma-Aldrich, St. Louis, Missouri, USA) and exosomes, and then seeded into a corning transwell chamber (pore size: 8.0 μm; Corning Brand, New York, New York State, USA). 24 h later, migrated cells were washed, fixed, and stained. Four photographs taken at 100× magnification was analyzed using ImageJ (National Institute of Health, Maryland, USA).

**Tube formation**

A 48-well plate and 200-μl tips were pre-cooled in the 4°C refrigerator. Then, endothelial cells were seeded in the 48-well plate pre-coated with 50 μl Matrigel basement membrane matrix (Corning Brand, New York, Corning, New York State, USA). Cells began to form tubes immediately after the transplantation and photographs were taken after 6 h (200× magnification). The total lengths of the formed tubes were calculated by using ImageJ.

**Nerite Outgrowth**

PC12 cells were seeded on a 6-well plate with a moderate density, and pre-treated with LPS, miRNA mimics, and inhibitors. Then, 50 ng/mL neuronal growth factor (NGF) (Proteintech, Chicago, Illinois, USA) was used to stimulate PC12 cells with a 2.5% FBS for 3-5 days. During stimulation, culture was replaced every other two days. Four photographs were casually taken (200× magnification) and fifty PC12 cells with neurites were included to calculate the average length of the extended neurites. The average nerite length was analyzed by ImageJ. For primary cells, neurons were subjected to Exo and Exo-K before treated with LPS for 12h. Then, four photographs were randomly captured by an inverted fluorescence microscope.

**Transfection of Plasmids, miRNAs and siRNAs**

The plasmids containing cDNA clones of Cbl and Cblb genes were constructed and purchased from Guangzhou RiboBio Co., Ltd. (Guangzhou, China). These cDNA fragments containing coding sequence (CDS) of the Cbl and Cblb gene were cloned into pcDNA3.0 vector (Life Technologies, Boston, MA, USA). The PCR fragment was verified by DNA sequencing, RT-QPCR and western blot. Specific miRNAs’ sequences obtained from TargetScan Human 7.2 and siRNA of Cblb and Cbl genes were synthesized by RiboBio as well. Plasmids, mimics and antisense sequences of miRNAs, and SiRNAs were transfected by using lipo-3000 reagent (Gibco, Carlsbad, California, USA). Core sequences, SiRNA sequences of Cbl and Cblb, and miRNA sequences used in this experiment are provided in **Additional file 3 and 4.**

**RT-QPCR**

Nuclear and cytoplasmic RNAs were extracted via the Trizol reagent (Invitrogen, Carlsbad, California, USA) and the concentration of total RNA was detected by NaroDrop (Thermo Fisher, Boston, MA, USA). The complementary cDNA of target mRNA was synthesized by reverse transcription following the manufacturer’s protocol. In order to validate RT-QPCR of miRNA, we added a cervical loop to miRNA, then reversed miRNA and amplified cDNA of miRNA using primers particularly designed for each miRNA. Quantitative reverse transcription polymerase chain reactions (RT-QPCR) were carried out by using the SYBR Green PCR kit (Gene Star, Beijing, China). Normal polymerase chain reactions (PCR) was conducted using a common PCR instrument. The relative expression was calculated using the following equation: relative gene expression= 2^−^ ^(ΔCtsample−ΔCtcontrol)^. The primer sequences are listed in **Additional file 5**.

**Luciferase reporter gene assay**

We amplified the genomic DNA fragment of Cbl and Cblb containing the predicted miRNAs binding sites (wild type, WT) and mutant sites (mutant type, MT) and cloned into PGL3-report Dual-luciferase miRNA Target Expression Vector. The PGL3-report and pRL-TK vectors were respectively co-transfected with miRNA mimics or inhibitors by using lipo-3000 reagent into HEK293 cells. Following transfection, luciferase activity was detected by Dual-Luciferase Reporter Assay System (Promega, Madison, Wisconsin, USA).

**Western blot**

Proteins from cells and spinal cords (5 mm length of spine cord centered the injured site) were extracted by using RIPA buffer and quantified by BCA Kit. 10-30 μg of lysate was loaded on each lane. Primary antibodies against IL-6 (GeneTex, San Antonio, Texas, USA), IL-1 (GeneTex, San Antonio, Texas, USA), TNF-α (GeneTex, San Antonio, Texas, USA), Akt (CST, Boston, MA, USA), p-Akt (CST, Boston, MA, USA), Erk (CST, Boston, MA, USA), p-Erk (CST, Boston, MA, USA), NF-H (CST, MA, USA), Neu-N (CST, Boston, MA, USA), β-tubulin-Ⅲ (CST, Boston, MA, USA), TrkA (Abcam, Cambridge, USA), Cblb (CST, Boston, MA, USA) and Cbl (CST, Boston, MA, USA) were used. Corresponding secondary antibodies conjugated with horseradish peroxidase (HRP) were used to bind to primary antibodies. Protein bands were visualized using enhanced chemiluminescence (ECL; Thermo Fisher, Boston, MA, USA) and band intensity was analyzed using ImageJ.

**Co-precipitation (Co-IP)**

Cells were pre-transfected with siRNAs and plasmids expressing GFP-tagged Cblb and Cbl for 24-36 h, and then the cells were lysed and total proteins were extracted. TrkA monoclonal antibody was used for IP testing, subsequently, western blot was used to detect Cblb and Cbl protein expressions.

**SCI models**

For all rats, anesthesia was conducted using 0.1% pentobarbital with 0.01 mL/g. Laminectomy of vertebras from T9-T11 was performed to expose the spinal cord. Thereafter, an artery clamp (35 g for 30s) was applied to trigger acute trauma to the exposed T10 segment (3 mm depth for 30s). Successful models would have you seen these behaviors of rats: hemorrhage and edema encompassing the injured site, flicking of bilateral hind limbs, swaying of the tail. A laminectomy was performed in sham group without damage. 30 min post SCI, 200 μg exosomes (Exo group) and exosomes with the inhibition of miR-199a-3p/145-5p were intravenously injected. 24 h later, 200 μg exosomes were again injected. The rats in the SCI group accepted equivalent PBS injection. Then, all rats were exposed to heated lamp for 2h following surgical procedures. Within two weeks, we massaged the urinary bladders of rat to help urinate and give 10^5^IU penicillin to rats with urinary system infection till the urine became clear.

**BBB rating scale**

BBB scores were evaluated at 1, 3, 5, 7, 14, 21, 28, and 35-day post injury (DPI) by two independent researchers who were blinded to groups in an open place. The locomotor score of each rat, ranging from 0-21, was recorded.

**Magnetic Resonance Imaging (MRI)**

At week 5, MRI (Magnetic Resonance Imaging) was conducted with a 7 T bore scanner (Bruker Biospec, Ettlingen, Germany). Rats were anesthetized with pentobarbital and fixated on the pad. Sagittal and axial T2-weighted anatomical images were scanned using RARE (rapid acquisition relaxation-enhanced) sequence. The RARE acquisition parameters were: 256 × 256 matrix, slice thickness: 1 mm, intersection gap: 1 mm, echo time/repetition time: 27/3000 ms, RARE factor: 16, and flip angle: 90°

**Tunnel staining**

First, the rat was sacrificed by CO_2_ suffocation and perfused by 4% paraformaldehyde via ventriculus sinister. Then, about 1.5 cm long spine cord was removed intactly centering the injured site and embedded into paraffin. Briefly, after deparaffinization and rehydration, 5 μm longitudinal sections were stained using an in-situ cell death detection kit (Roche, Mannheim, Germany) following the manufacturer’s instructions. The intact slice was scanned by the [laser](javascript:;) [scanning](javascript:;) [confocal](javascript:;) [microscope](javascript:;) (Leica, Heidelberg, Germany).

**Immunofluorescence**

***Cell***

Cells were first fixed by 4% paraformaldehyde and then blocked by Tris Buffered Saline Tween (TBST) containing 5% bovine serum albumin (BSA). Primary antibodies against NF-H and Neu-N, and β-tubulin-Ⅲ, vimentine (CST, Boston, MA, USA), CD31 (CST, Boston, MA, USA), GFAP, and NF-H were used to hybridize antigens. Then, fluorescein-conjugated secondary antibodies (Thermo Fisher, Boston, MA, USA) were used to hybridize primary antibodies. Photographs were taken by using an inverted fluorescence Microscope or the laser scanning confocal microscope.

***Tissues***

The 5-μm-paraffin sections were stained with primary antibodies against MAP2 (CST, Boston, MA, USA), Neu-N, GFAP (Abcam, Cambridge, UK)**.** Subsequently the sections were incubated with fluorescein-conjugated secondary antibodies (Thermo Fisher, Boston, MA, USA). The sections were finally stained with Hochest 33342 (Beyotime, Shanghai, China). Images were scanned using [laser](javascript:;) [scanning](javascript:;) [confocal](javascript:;) [microscope](javascript:;) and analyzed by ImageJ.

**HE staining**

Briefly, after deparaffinization and rehydration, 5 μm longitudinal sections were stained with hematoxylin and eosin solution. The mounted slides were then scanned by using [laser](javascript:;) [scanning](javascript:;) [confocal](javascript:;) [microscope](javascript:;). The lesion size of spine cord was calculated by ImageJ software.

**Reference**

[1] Franzen R, Martin D, Daloze A, Moonen G, Schoenen J. Grafts of Meningeal Fibroblasts in Adult Rat Spinal Cord Lesion Promote Axonal Regrowth. Neuroreport. 1999;10(7):1551-56.

[2] Schildge S, Bohrer C, Beck K, Schachtrup C. Isolation and Culture of Mouse Cortical Astrocytes. J Vis Exp. 2013(71).

[3] Wang J M, Chen A F, Zhang K. Isolation and Primary Culture of Mouse Aortic Endothelial Cells. J Vis Exp. 2016(118).

[4] Malin S A, Davis B M, Molliver D C. Production of Dissociated Sensory Neuron Cultures and Considerations for their Use in Studying Neuronal Function and Plasticity. Nat Protoc. 2007;2(1):152-60.

[5] Junghans U, Koops A, Westmeyer A, Kappler J, Meyer H E, Müller H W. Purification of a Meningeal Cell-Derived Chondroitin Sulphate Proteoglycan with Neurotrophic Activity for Brain Neurons and its Identification as Biglycan. Eur J Neurosci. 1995;7(11):2341-50.

[6] Wang Y, Wang J, Li Y, Wang S, Zhu X. Platelet-Rich Plasma Protects HUVECs Against oX-LDL-induced Injury. Open Med (Wars). 2018; 13:41-52.
