# Supplementary material for: Umbilical mesenchymal stem cell-derived exosomes facilitate spinal cord functional recovery through the miR-199a-3p/145-5p-mediated NGF/TrkA signaling pathway in rats
Source: Stem Cell Res Ther. 2021 Feb 12;12:117. doi: 10.1186/s13287-021-02148-5 (PMC7879635; doi:10.1186/s13287-021-02148-5)
Supplement: Supplementary file 3 — Additional file 3. SiRNA and miRNA sequences. [file 13287_2021_2148_MOESM3_ESM.docx]

| **Item** | **Sequence (5’-3’)** |
| --- | --- |
| **miR-199a-3p** |  |
| Mimic | AAGUAGUCUGCACAUUGGUUA |
| Inhibitor | UAACCAAUGUGCAGACUACUU |
| **miR-145-5p** |  |
| Mimic | GUCCAGUUCCCAGGAAUCCCU |
| Inhibitor | AGGGAUUCCUGGGAACUGGAC |
| **SiRNA of Cblb** |  |
| #001 | GTGAACCTACACCTCATGA |
| #002 | CAGAAATCAAGGCGATCTT |
| #003 | CCAGAAATTCATCACAGAA |
| **SiRNA of Cbl** |  |
| #001 | GGAACATCCTGCAGACAAT |
| #002 | CCAGAAGTTCATCCACAAA |
| #003 | CCAGGAACAATATGAACTA |
| **Scramble sequence** | ACAGGUCCCUGCACAUUGGUUA |

**Additional file 3. SiRNA and miRNA sequences**
