# Supplementary material for: Umbilical mesenchymal stem cell-derived exosomes facilitate spinal cord functional recovery through the miR-199a-3p/145-5p-mediated NGF/TrkA signaling pathway in rats
Source: Stem Cell Res Ther. 2021 Feb 12;12:117. doi: 10.1186/s13287-021-02148-5 (PMC7879635; doi:10.1186/s13287-021-02148-5)
Supplement: Supplementary file 4 — Additional file 4. Coding Sequence of Cbl and Cblb Genes. [file 13287_2021_2148_MOESM4_ESM.docx]

**Additional file 4. Coding Sequence of Cbl and Cblb Genes**

| **Gene** | **Sequence** |
| --- | --- |
| **Cblb**  **[NM_133601.1 (complete CDS)]** | 45 atggca aattctatga  61 atggcagaaa tcctggtggt cgaggaggaa acccccgcaa aggtcgaatt ttggggatta  121 ttgatgccat tcaggatgca gttggacccc caaagcaagc tgcagctgac cgcaggacag  181 tggagaagac ttggaaactc atggacaaag tggtaagact gtgccaaaat ccgaaacttc  241 agttgaaaaa cagcccacca tatatcctcg acattttacc tgatacgtat cagcatttgc  301 ggcttatatt gagtaagtat gacgacaacc agaagctggc tcaactgagc gagaatgagt  361 actttaaaat ctacatcgac agtctcatga agaagtcaaa gcgagcgatc cggctcttca  421 aagaaggcaa ggagaggatg tacgaggagc agtcgcagga cagacggaat ctcacaaagc  481 tgtcccttat cttcagtcac atgctggcag aaatcaaggc gatctttccc aatggccagt  541 tccagggaga taacttccgg atcaccaaag cagatgctgc cgaattctgg aggaagtttt  601 ttggagacaa aactatcgta ccatggaaag tcttcagaca gtgcctgcat gaggtccatc  661 agatcagctc tggcctggag gccatggctc tgaagtcaac cattgactta acttgtaatg  721 attacatctc cgtgtttgaa tttgatattt ttaccaggct atttcagccc tggggctcta  781 ttttacggaa ttggaacttc ttagctgtga cacacccggg gtacatggca tttctcacat  841 atgatgaagt taaagctcga ctacagaaat acagcaccaa gcctggaagc tacattttcc  901 ggttaagctg cactcggctg ggacaatggg ccattggcta tgtgactggg gacggcaata  961 tcctacagac catacctcat aacaagcccc tgttccaagc cctgattgat ggtagcaggg  1021 aaggctttta cctttatcca gatggacgaa gctataaccc tgatttaacc ggattatgtg  1081 aacctacacc tcatgatcat ataaaagtta cacaggagca atatgaactg tattgtgaaa  1141 tgggctccac ttttcagctg tgcaagatct gtgcagagaa tgacaaagat gtcaagatcg  1201 agccttgtgg gcatctcatg tgcacttcgt gccttaccgc gtggcaggag tctgatggcc  1261 aaggctgccc cttctgtcgc tgtgagataa aaggaaccga acctatcatc gtggatccct  1321 ttgaccccag agacgaaggc tccaggtgct gcagcatcat cgaccctttc agcatcccca  1381 tgctcgactt ggatgatgac gatgatcgag aggagtctct gatgatgaac cggctggcga  1441 gtgttcgcaa gtgcacagac aggcagaact cgccagtcac atcgccagga tcctcacccc  1501 ttgcccagag aagaaagcct cagccagacc ctctccagat cccccacctc agcctgccac  1561 cagtgcctcc ccgcctggac ctcattcaga aaggcatcgt gcgctctccc tgtggcagcc  1621 ccacgggctc cccgaagtct tctccatgca tggttagaaa acaagacaaa ccactcccag  1681 caccccctcc tcccttgcga gatcctccgc ctccaccaga gcggcctccg ccaatcccgc  1741 ctgacagtag actgagcaga cacttccacc acggagagag tgtgccttcc agggaccagc  1801 caatgcctct tgaagcctgg tgccctcggg atgccttcgg gactaatcag gtgatgggat  1861 gtcgcatcct aggggatggc tctccaaagc ctggcgtcac agcaaactcc aacttaaatg  1921 gacgtcacag tcgaatgggc tctgaccagg ttcttatgag gaaacacaga cgccacgatt  1981 tgccttcaga aggcgccaag gtcttttcca atggacacct tgcccctgaa gaatacgacg  2041 ttcctcctcg gctttcccct cctcctccag tcactgccct tctccctagc ataaagtgta  2101 ctggtccaat agcaaattgt ctctccgaga aaacaagaga cacagtagaa gaagatgatg  2161 atgaatacaa gattccttca tcccatcctg tttccctgaa ttcacaacca tctcattgtc  2221 ataatgtcaa acctcctgtt cggtcttgtg ataatggtca ctgtatactg aatggaactc  2281 atggtacgcc ttcagagatg aagaaatcaa acatcccaga tttaggcatc tatttgaagg  2341 gtgaagatgc ttttgatgcc ctccccccat cccttcctcc tcccccacct cctgcaagac  2401 atagtctcat cgagcattca aaacctccag gctccagtag ccggccttcc tcaggacagg  2461 accttttcct tcttccttca gatccctttt ttgacccagc aagtggccaa gttccattgc  2521 ctccggccag gagagcacca ggagatggtg tcaaatccaa cagagcctcc caggactatg  2581 accagctccc ttcatcttcc gatggttcgc aagcaccagc tagacccccc aaaccacgac  2641 cccgaaggac tgcaccagaa attcatcaca gaaagcccca tgggcccgag gcggcactgg  2701 aaaatgtgga tgcgaaaatt gcaaaactca tgggagaggg gtatgccttt gaagaggtga  2761 agagagcctt agagatcgcc cagaataacc tggaagtggc caggagcata cttcgagaat  2802 tcgccttccc tcctcccgtc tcgccacgtc tcaatctata g |
| **Cbl**  [**XM_006242965.3 (complete CDS)**] | 153 atggccgg caacgtgaag aagagctcgg  181 gcgccggcgg cagcggctct gggggctccg gagcgggcgg cctgatcggg ctcatgaagg  241 acgccttcca gccgcaccac caccaccacc accacctcag cccgcaccct ccctgcacgg  301 tggacaagaa gatggtggag aagtgctgga agctcatgga caaggtggtg cggttgtgtc  361 aaaacccaaa gctggctctc aagaacagcc caccgtatat cttagacctg ctgcctgaca  421 cctaccagca cctccgcact gtcctgtcga gatatgaggg gaagatggag acgcttggag  481 agaatgagta tttcagggtg ttcatggaga atttgatgaa gaaaactaag cagaccatca  541 gcctcttcaa ggagggaaaa gaaaggatgt atgaggagaa ttcccagcct aggcgaaacc  601 taaccaagtt atccctgatc ttcagccaca tgctggcaga actgaaaggc atctttccaa  661 gcggactctt ccaaggagac acttttcgga tcactaaagc agacgctgca gagttctgga  721 gaaaagcttt tggggaaaag acgatagtcc cttggaagag ctttcgacag gccctgcatg  781 aagtgcatcc catcagttct gggctggagg ccatggcttt gaagtccact attgatctga  841 cctgcaatga ttatatttct gtttttgaat ttgatatttt tacacggctg tttcagccct  901 ggtcctcttt gctcagaaat tggaacagcc ttgctgtaac tcaccctggc tacatggctt  961 tcctgacgta tgatgaagtg aaagctcggc tccagaagtt catccacaaa cccggcagtt  1021 acatctttcg gctgagctgt actcgtttgg gtcagtgggc tattgggtac gttacagctg  1081 atgggaacat cctgcagaca atcccacaca ataaaccgct cttccaagca ctgattgatg  1141 gcttcaggga aggcttctat ttgtttcctg atggaagaaa tcaaaatcct gacctgacag  1201 gtttatgtga accaacccct caagatcata tcaaagtcac ccaggaacaa tatgaactat  1261 actgtgaaat gggctccaca tttcaactat gtaagatatg tgctgagaat gataaggatg  1321 tgaagattga gccctgtgga cacctcatgt gcacgtcctg cctcacatcg tggcaggaat  1381 cagaaggtca aggctgtcct ttttgccgat gtgaaatcaa aggtactgag cccatcgtgg  1441 tggatccatt tgaccccaga ggcagtggca gtctattaag gcaaggagcg gaaggtgctc  1501 cttccccaaa ttatgatgat gatgatgatg aacgagctga tgattctctc ttcatgatga  1561 aggagttggc aggtgccaag gcttcttcta gctcccttca taaggacaaa ccattgccaa  1621 tacctcctac gcttcgagat cttcctccac caccccctcc agaccggcct tactctgttg  1681 gaacagaaac aaggcctcag agacgccccc tgccttgtac tccaggcgac tgtccatcta  1741 gagacaaact gccccctgtc ccctctagcc gcccagggga ctcatggttg tctcggccaa  1801 tccctaaagt accagtagcg actccaaacc ctggtgatcc ttggaatggg agagaattga  1861 ccaatcggca ctcacttcca ttctcattgc cctcacaaat ggaacccaga gcagatgttc  1921 caaggcttgg aagcacattt agtctggata cctccacgac tatgaatagc agcccagtag  1981 caggtccaga gagtgagcac ccaaaaatca agccttcctc atccgccaat gccatttact  2041 ctctggctgc caggcctctt cctgtgccaa aactgccacc tggggagcaa ggggacagtg  2101 aagaagacac ggaatatatg actcccacgt ctaggcctgt aggggttcag aagccagagc  2161 ccaaacggcc tgcagagaca gcgcagagtt cacgagcctg tgactgtgac cagcagattg  2221 acagctgcac atacgaagca atgtataaca ttcagtccca agcactctcc gtagcagaga  2281 acagtgcctg tggagaaggg aatttggcca cagcccacac cagtactggc ccggaggagt  2341 cagaaaacga ggatgatggc tatgatgtac cgaagcctcc tgtgccagct gtactggccc  2401 gccggaccct gtctgacatc tccaatgcca gctcctcctt tggctggtta tctttggatg  2461 gcgaacccac aaacttcaat gagggttccc aagttcctga gcggcccccc aaaccattcc  2521 ctcggagaat caactcagaa cgaaaagcaa gtagctatca acaaggcgga ggtgccatgg  2581 ctaaccctgc agccgccgcc ccctcaccgc agctctcaag cgagattgaa cgcctcatga  2641 gtcagggcta ttcctaccag gacattcaga aagcgttggt cattgcccac aacaacattg  2701 agatggccaa aaacatcctc cgggaattcg tttctatttc ttctcctgcc catgtagcca  2761 cctag |
