# Supplementary material for: Umbilical mesenchymal stem cell-derived exosomes facilitate spinal cord functional recovery through the miR-199a-3p/145-5p-mediated NGF/TrkA signaling pathway in rats
Source: Stem Cell Res Ther. 2021 Feb 12;12:117. doi: 10.1186/s13287-021-02148-5 (PMC7879635; doi:10.1186/s13287-021-02148-5)
Supplement: Supplementary file 5 — Additional file 5. Primers of target genes. [file 13287_2021_2148_MOESM5_ESM.docx]

| **Gene** | **Forward primer (5’-3’)** | **Reverse primer (5’-3’)** |
| --- | --- | --- |
| **NF-H** | GGAGTGGTTCCGAGTGAGATTG | CCTTGGTGCTTTTCAGTGCCT |
| **Neu-N** | GGCAAATGTTCGGGCAATT | TCGGTCAGCATCTGAGCTAGTT |
| **β-tubulin-Ⅲ** | CCCGTTTTAGCCACCTTTGTATT | CCCTCCAAATATAAACACAACCC |
| **Cbl** | TGACCAATCGGCACTCACTT | GGCTGCTATTCATAGTCGTGGA |
| **Cblb** | GGGCTCTATTTTACGGAATTGG | GGCTTGGTGCTGTATTTCTGTAGT |
| **β-actin** | TCAAGATCATTGCTCCTCCTGAG | ACATCTGCTGGAAGGTGGACA |

**Additional file 5. Primers of target genes**

NF-H, neuronal filament-heavy.
