# Supplementary material for: Umbilical mesenchymal stem cell-derived exosomes facilitate spinal cord functional recovery through the miR-199a-3p/145-5p-mediated NGF/TrkA signaling pathway in rats
Source: Stem Cell Res Ther. 2021 Feb 12;12:117. doi: 10.1186/s13287-021-02148-5 (PMC7879635; doi:10.1186/s13287-021-02148-5)
Supplement: Supplementary file 6 — Additional file 6. Outcome of miRNA sequencing. [file 13287_2021_2148_MOESM6_ESM.docx]

| **Additional file 6. Outcome of miRNA sequencing** | | | |
| --- | --- | --- | --- |
| miRNA | Sample 1 | Sample 2 | Sample 3 |
| hsa-miR-21-5p | 159223 | 176767.1 | 167067.6 |
| hsa-miR-24-3p | 102320.8 | 98448.27 | 94028.34 |
| hsa-miR-22-3p | 61187.06 | 59249.28 | 57861.17 |
| hsa-miR-26a-5p | 46544.34 | 52092.82 | 45509.93 |
| hsa-miR-146a-5p | 43691.26 | 40471.19 | 36702.49 |
| hsa-miR-221-3p | 35744.16 | 32458.3 | 29144.96 |
| hsa-let-7a-5p | 30058.97 | 31633.51 | 24939.87 |
| hsa-miR-29a-3p | 29344.13 | 28354.69 | 27921.7 |
| novel_115 | 23141.15 | 7951.877 | 21110.23 |
| hsa-miR-100-5p | 22721.89 | 20725.95 | 19116.69 |
| hsa-miR-222-3p | 21025.98 | 18825.54 | 14603.97 |
| hsa-miR-143-3p | 18640.38 | 18039.17 | 16607.2 |
| hsa-miR-125b-5p | 17787.18 | 21602.71 | 21606.8 |
| hsa-let-7f-5p | 17481.12 | 18436.88 | 16883.34 |
| hsa-miR-148a-3p | 16001.12 | 19693.27 | 19465.5 |
| hsa-let-7i-5p | 15835.52 | 15745.58 | 12273.73 |
| hsa-miR-199a-3p | 15544.13 | 17817.72 | 17585.8 |
| hsa-miR-122-5p | 12856.66 | 14136.67 | 10384.34 |
| hsa-miR-16-5p | 11892.36 | 10894 | 8284.221 |
| hsa-miR-27a-3p | 11491.96 | 11006.99 | 11314.5 |
| hsa-miR-23a-3p | 11028.68 | 12161.69 | 12307.64 |
| hsa-miR-181a-5p | 8808.689 | 9820.648 | 10304.41 |
| hsa-miR-199b-3p | 7768.92 | 8907.73 | 8792.902 |
| hsa-miR-495-3p | 7207.109 | 6573.462 | 5721.442 |
| hsa-miR-126-3p | 7150.509 | 9350.631 | 8611.23 |
| hsa-miR-31-5p | 6469.208 | 7447.965 | 7232.949 |
| hsa-miR-381-3p | 6236.518 | 6367.829 | 5399.278 |
| novel_97 | 6064.621 | 4020.003 | 3846.592 |
| hsa-miR-92a-3p | 5947.228 | 5233.461 | 4246.269 |
| hsa-miR-218-5p | 5922.072 | 5355.484 | 3635.853 |
| hsa-miR-26b-5p | 4767.006 | 5466.21 | 5418.656 |
| hsa-miR-320a | 4465.137 | 3190.694 | 2732.34 |
| hsa-miR-451a | 4448.367 | 4578.148 | 5953.981 |
| hsa-miR-151a-3p | 4423.211 | 4483.241 | 3543.806 |
| hsa-miR-128-3p | 4402.248 | 3690.087 | 3119.906 |
| hsa-miR-199a-5p | 4270.181 | 4264.05 | 3449.337 |
| hsa-miR-27b-3p | 3982.986 | 4001.925 | 4125.155 |
| hsa-miR-181b-5p | 3651.77 | 3208.771 | 2952.768 |
| hsa-miR-127-3p | 3458.909 | 3642.633 | 3505.049 |
| hsa-miR-423-5p | 3314.264 | 3421.183 | 2824.387 |
| novel_107 | 3280.723 | 6756.497 | 11602.75 |
| hsa-miR-125a-5p | 3196.871 | 2917.27 | 2858.299 |
| hsa-miR-7-5p | 3161.233 | 2300.373 | 1729.513 |
| novel_25 | 2941.121 | 0 | 1458.217 |
| hsa-miR-22-5p | 2928.543 | 3109.345 | 2870.41 |
| hsa-miR-23b-3p | 2765.031 | 3170.356 | 3415.425 |
| hsa-miR-155-5p | 2714.72 | 2548.939 | 1494.551 |
| hsa-miR-99a-5p | 2695.853 | 3125.162 | 2633.026 |
| hsa-let-7g-5p | 2693.757 | 2404.319 | 2245.46 |
| hsa-miR-423-3p | 2693.757 | 2022.43 | 1635.044 |
| hsa-miR-369-3p | 2609.904 | 2881.115 | 3158.662 |
| hsa-miR-425-5p | 2595.23 | 3009.918 | 2480.422 |
| novel_89 | 2559.593 | 2976.022 | 3294.31 |
| hsa-miR-191-5p | 2396.081 | 3052.852 | 3250.709 |
| novel_83 | 2393.984 | 0 | 0 |
| hsa-miR-30d-5p | 2352.058 | 2356.865 | 2046.832 |
| hsa-miR-99b-5p | 2295.458 | 2153.492 | 1877.272 |
| novel_100 | 2280.784 | 4822.196 | 8516.761 |
| hsa-miR-10a-5p | 2196.931 | 1791.941 | 1480.017 |
| hsa-miR-30c-5p | 2142.427 | 2133.155 | 1714.979 |
| hsa-miR-10b-5p | 2035.516 | 1907.185 | 1748.891 |
| hsa-miR-409-3p | 1999.878 | 1459.765 | 1254.745 |
| hsa-miR-411-5p | 1991.493 | 1852.952 | 1717.401 |
| hsa-miR-543 | 1901.352 | 1938.821 | 1613.243 |
| hsa-miR-29b-3p | 1836.366 | 1631.502 | 1455.794 |
| hsa-miR-30a-5p | 1832.174 | 1938.821 | 2058.944 |
| novel_36 | 1823.788 | 1204.419 | 1211.143 |
| hsa-miR-103a-3p | 1821.692 | 1864.251 | 1821.56 |
| hsa-miR-370-3p | 1781.862 | 1710.591 | 1383.126 |
| hsa-miR-25-3p | 1716.877 | 1410.052 | 1007.671 |
| novel_55 | 1572.231 | 1545.633 | 1569.642 |
| hsa-miR-487b-3p | 1565.943 | 1534.335 | 1480.017 |
| hsa-miR-214-3p | 1547.076 | 1437.168 | 1288.657 |
| hsa-miR-337-3p | 1544.979 | 1319.664 | 1172.387 |
| hsa-miR-493-5p | 1526.113 | 1378.416 | 1436.416 |
| novel_137 | 1526.113 | 0 | 0 |
| hsa-let-7b-5p | 1463.223 | 1599.866 | 1182.076 |
| hsa-miR-186-5p | 1396.142 | 1597.607 | 1361.325 |
| novel_69 | 1370.986 | 0 | 1908.762 |
| hsa-miR-654-3p | 1293.422 | 908.3986 | 874.4456 |
| hsa-miR-379-5p | 1289.23 | 1082.395 | 1000.405 |
| novel_98 | 1268.267 | 0 | 0 |
| hsa-miR-154-5p | 1264.074 | 1181.822 | 879.2902 |
| hsa-miR-329-3p | 1257.785 | 876.7628 | 789.6656 |
| hsa-miR-369-5p | 1255.689 | 1312.885 | 1133.63 |
| hsa-miR-1307-3p | 1243.111 | 930.9956 | 760.5981 |
| hsa-miR-150-5p | 1215.859 | 1145.667 | 1121.519 |
| hsa-miR-126-5p | 1199.089 | 1265.431 | 1334.68 |
| hsa-miR-382-5p | 1196.992 | 1369.377 | 1327.413 |
| novel_122 | 1163.451 | 0 | 0 |
| hsa-miR-152-3p | 1146.681 | 1073.357 | 1136.053 |
| novel_81 | 1113.14 | 0 | 0 |
| hsa-miR-193a-5p | 1073.31 | 921.9568 | 930.1582 |
| hsa-miR-98-5p | 1048.154 | 994.2671 | 801.777 |
| hsa-miR-6087 | 1016.71 | 680.1691 | 593.4603 |
| hsa-miR-134-5p | 1012.517 | 804.4525 | 733.953 |
| hsa-miR-15b-5p | 987.3614 | 1039.461 | 833.2667 |
| hsa-miR-196b-5p | 974.7835 | 906.1389 | 620.1055 |
| hsa-miR-378a-3p | 962.2057 | 892.5807 | 746.0644 |
| hsa-miR-335-5p | 943.3389 | 779.5958 | 1126.363 |
| hsa-let-7e-5p | 941.2426 | 1043.981 | 818.733 |
| hsa-miR-34a-5p | 922.3758 | 1041.721 | 772.7095 |
| hsa-miR-539-5p | 882.5459 | 1123.07 | 859.9119 |
| hsa-miR-431-5p | 874.1607 | 698.2467 | 615.2609 |
| hsa-miR-493-3p | 853.1976 | 897.1001 | 828.4221 |
| hsa-miR-532-5p | 838.5234 | 892.5807 | 794.5101 |
| hsa-miR-196a-5p | 836.4271 | 714.0646 | 513.5248 |
| hsa-miR-320b | 830.1382 | 646.2736 | 731.5307 |
| hsa-miR-494-3p | 794.501 | 781.8555 | 716.9969 |
| hsa-miR-146a-3p | 786.1157 | 666.6109 | 561.9706 |
| hsa-let-7d-5p | 775.6342 | 919.6971 | 709.7301 |
| hsa-miR-101-3p | 748.3822 | 797.6734 | 651.5952 |
| hsa-miR-374a-5p | 746.2859 | 756.9988 | 743.6421 |
| novel_71 | 687.5892 | 795.4137 | 1337.102 |
| hsa-miR-139-5p | 658.2409 | 827.0495 | 731.5307 |
| hsa-miR-222-5p | 635.1815 | 345.7338 | 440.8562 |
| hsa-miR-125b-1-3p | 607.9295 | 598.82 | 392.4105 |
| hsa-miR-30b-5p | 605.8332 | 745.7004 | 830.8444 |
| hsa-miR-376a-3p | 599.5443 | 506.1724 | 460.2345 |
| hsa-miR-374b-5p | 593.2553 | 653.0527 | 607.994 |
| novel_64 | 586.9664 | 0 | 0 |
| hsa-miR-224-5p | 580.6775 | 476.7963 | 327.0087 |
| hsa-miR-142-3p | 566.0033 | 596.5603 | 566.8152 |
| hsa-miR-93-5p | 563.907 | 653.0527 | 423.9002 |
| novel_150 | 549.2329 | 262.125 | 358.4985 |
| hsa-let-7c-5p | 528.2698 | 657.5721 | 368.1876 |
| hsa-miR-574-3p | 526.1735 | 451.9396 | 472.346 |
| hsa-miR-107 | 524.0772 | 415.7844 | 423.9002 |
| hsa-miR-486-5p | 519.8845 | 451.9396 | 380.2991 |
| hsa-miR-361-5p | 501.0178 | 427.0829 | 356.0762 |
| novel_66 | 492.6325 | 702.7661 | 0 |
| hsa-miR-615-3p | 486.3436 | 370.5905 | 402.0996 |
| hsa-miR-30e-5p | 475.8621 | 397.7069 | 394.8328 |
| hsa-miR-320c | 473.7657 | 375.1099 | 445.7008 |
| hsa-miR-92b-3p | 471.6694 | 314.098 | 261.607 |
| hsa-miR-323a-3p | 471.6694 | 363.8114 | 368.1876 |
| hsa-miR-376c-3p | 463.2842 | 433.862 | 419.0556 |
| hsa-miR-145-5p | 444.4174 | 386.4084 | 455.39 |
| novel_109 | 442.3211 | 0 | 0 |
| hsa-miR-7704 | 433.9359 | 305.0592 | 288.2522 |
| novel_42 | 427.647 | 185.2952 | 363.343 |
| hsa-miR-655-3p | 421.358 | 388.6681 | 482.0351 |
| hsa-miR-455-5p | 412.9728 | 451.9396 | 295.519 |
| novel_74 | 408.7802 | 413.5247 | 605.5717 |
| novel_30 | 406.6839 | 0 | 0 |
| hsa-miR-337-5p | 400.3949 | 418.0441 | 394.8328 |
| hsa-miR-148b-3p | 387.8171 | 420.3038 | 373.0322 |
| hsa-miR-140-3p | 373.1429 | 388.6681 | 394.8328 |
| hsa-miR-28-3p | 371.0466 | 230.4892 | 215.5835 |
| novel_94 | 364.7577 | 0 | 0 |
| hsa-miR-24-2-5p | 362.6614 | 377.3696 | 358.4985 |
| hsa-miR-154-3p | 356.3725 | 433.862 | 494.1465 |
| hsa-miR-30a-3p | 347.9872 | 381.889 | 302.7859 |
| hsa-miR-19b-3p | 347.9872 | 370.5905 | 300.3636 |
| hsa-miR-432-5p | 343.7946 | 332.1756 | 259.1847 |
| hsa-miR-335-3p | 335.4094 | 210.1519 | 179.2492 |
| hsa-miR-28-5p | 331.2168 | 334.4353 | 273.7184 |
| hsa-miR-503-5p | 329.1205 | 210.1519 | 150.1818 |
| novel_73 | 327.0241 | 221.4504 | 218.0058 |
| novel_1 | 324.9278 | 361.5517 | 271.2961 |
| hsa-miR-137 | 322.8315 | 284.722 | 237.3841 |
| novel_52 | 322.8315 | 0 | 264.0293 |
| hsa-miR-106b-5p | 314.4463 | 298.2801 | 312.475 |
| novel_3 | 301.8684 | 329.9159 | 358.4985 |
| hsa-miR-411-3p | 301.8684 | 225.9698 | 205.8944 |
| hsa-miR-744-5p | 299.7721 | 320.8771 | 239.8064 |
| novel_4 | 289.2906 | 282.4623 | 227.695 |
| hsa-miR-142-5p | 289.2906 | 347.9935 | 399.6774 |
| hsa-miR-145-3p | 287.1943 | 244.0474 | 244.651 |
| hsa-miR-149-5p | 285.098 | 173.9968 | 159.8709 |
| hsa-miR-656-3p | 278.809 | 210.1519 | 247.0733 |
| hsa-miR-194-5p | 276.7127 | 307.3189 | 329.431 |
| hsa-miR-412-5p | 272.5201 | 280.2026 | 261.607 |
| hsa-miR-889-3p | 264.1349 | 286.9817 | 290.6744 |
| hsa-miR-484 | 264.1349 | 221.4504 | 193.783 |
| hsa-miR-3615 | 257.846 | 244.0474 | 259.1847 |
| hsa-miR-342-3p | 251.557 | 221.4504 | 232.5395 |
| novel_85 | 247.3644 | 164.958 | 0 |
| hsa-miR-424-3p | 243.1718 | 225.9698 | 220.4281 |
| hsa-miR-454-3p | 243.1718 | 207.8922 | 150.1818 |
| hsa-miR-345-5p | 243.1718 | 214.6713 | 198.6275 |
| hsa-miR-20a-5p | 241.0755 | 225.9698 | 169.5601 |
| novel_138 | 238.9792 | 155.9192 | 159.8709 |
| novel_103 | 234.7866 | 212.4116 | 205.8944 |
| novel_144 | 234.7866 | 0 | 0 |
| novel_5 | 232.6903 | 336.695 | 288.2522 |
| novel_91 | 232.6903 | 146.8804 | 92.04691 |
| hsa-miR-192-5p | 232.6903 | 239.528 | 227.695 |
| novel_2 | 230.5939 | 262.125 | 288.2522 |
| hsa-miR-1180-3p | 230.5939 | 135.5819 | 109.0029 |
| novel_61 | 224.305 | 323.1368 | 346.387 |
| novel_38 | 222.2087 | 115.2446 | 0 |
| hsa-miR-140-5p | 222.2087 | 198.8534 | 147.7595 |
| novel_35 | 218.0161 | 440.6411 | 663.7066 |
| hsa-miR-320d | 218.0161 | 180.7758 | 237.3841 |
| hsa-miR-106b-3p | 215.9198 | 271.1638 | 242.2287 |
| hsa-miR-132-3p | 209.6309 | 180.7758 | 196.2052 |
| hsa-miR-652-3p | 209.6309 | 250.8265 | 234.9618 |
| hsa-miR-193b-3p | 207.5346 | 183.0355 | 174.4047 |
| hsa-miR-185-5p | 203.3419 | 216.931 | 155.0264 |
| hsa-miR-382-3p | 201.2456 | 185.2952 | 171.9824 |
| hsa-miR-223-3p | 199.1493 | 210.1519 | 227.695 |
| hsa-miR-941 | 199.1493 | 135.5819 | 193.783 |
| hsa-miR-136-3p | 197.053 | 216.931 | 280.9853 |
| novel_58 | 194.9567 | 214.6713 | 203.4721 |
| hsa-miR-629-5p | 190.7641 | 135.5819 | 104.1583 |
| hsa-miR-15a-5p | 190.7641 | 205.6325 | 159.8709 |
| hsa-miR-17-5p | 190.7641 | 194.334 | 128.3812 |
| novel_106 | 190.7641 | 171.7371 | 193.783 |
| hsa-let-7d-3p | 188.6678 | 153.6595 | 147.7595 |
| novel_59 | 186.5715 | 0 | 0 |
| novel_47 | 186.5715 | 540.0678 | 888.9793 |
| novel_6 | 186.5715 | 235.0086 | 225.2727 |
| hsa-miR-660-5p | 182.3788 | 189.8146 | 227.695 |
| hsa-miR-31-3p | 180.2825 | 178.5161 | 159.8709 |
| hsa-miR-339-5p | 180.2825 | 173.9968 | 186.5161 |
| hsa-miR-197-3p | 180.2825 | 119.764 | 198.6275 |
| hsa-miR-410-3p | 178.1862 | 171.7371 | 157.4487 |
| hsa-miR-18a-5p | 176.0899 | 198.8534 | 174.4047 |
| hsa-miR-30e-3p | 176.0899 | 203.3728 | 155.0264 |
| hsa-miR-16-2-3p | 173.9936 | 144.6207 | 77.51318 |
| novel_102 | 173.9936 | 0 | 0 |
| novel_48 | 173.9936 | 155.9192 | 150.1818 |
| hsa-miR-490-5p | 173.9936 | 133.3222 | 77.51318 |
| hsa-miR-4488 | 171.8973 | 58.75215 | 62.97946 |
| hsa-miR-590-3p | 165.6084 | 183.0355 | 167.1378 |
| hsa-miR-361-3p | 163.5121 | 171.7371 | 125.9589 |
| hsa-miR-365a-3p | 163.5121 | 115.2446 | 133.2258 |
| hsa-miR-365b-3p | 163.5121 | 115.2446 | 133.2258 |
| novel_37 | 163.5121 | 153.6595 | 130.8035 |
| hsa-miR-99b-3p | 159.3195 | 180.7758 | 113.8475 |
| hsa-miR-136-5p | 159.3195 | 210.1519 | 162.2932 |
| hsa-miR-130a-3p | 155.1268 | 131.0625 | 128.3812 |
| novel_87 | 150.9342 | 0 | 0 |
| hsa-miR-340-5p | 148.8379 | 137.8416 | 140.4926 |
| hsa-miR-323b-3p | 146.7416 | 122.0237 | 113.8475 |
| hsa-miR-214-5p | 144.6453 | 137.8416 | 128.3812 |
| hsa-miR-1185-1-3p | 144.6453 | 135.5819 | 109.0029 |
| novel_62 | 144.6453 | 103.9461 | 157.4487 |
| novel_7 | 142.549 | 183.0355 | 171.9824 |
| hsa-miR-421 | 142.549 | 115.2446 | 79.93547 |
| hsa-miR-221-5p | 140.4527 | 135.5819 | 155.0264 |
| hsa-miR-758-3p | 140.4527 | 137.8416 | 135.6481 |
| hsa-miR-654-5p | 138.3564 | 178.5161 | 123.5366 |
| hsa-miR-450a-5p | 138.3564 | 126.5431 | 101.7361 |
| hsa-miR-146b-5p | 134.1638 | 164.958 | 135.6481 |
| novel_43 | 134.1638 | 67.79094 | 58.13489 |
| hsa-miR-328-3p | 132.0674 | 97.16702 | 87.20233 |
| hsa-miR-874-3p | 127.8748 | 65.53124 | 75.0909 |
| novel_8 | 127.8748 | 0 | 0 |
| novel_86 | 127.8748 | 101.6864 | 0 |
| hsa-miR-29a-5p | 127.8748 | 94.90732 | 77.51318 |
| novel_65 | 125.7785 | 0 | 0 |
| novel_129 | 125.7785 | 74.57004 | 0 |
| hsa-miR-331-5p | 123.6822 | 117.5043 | 87.20233 |
| novel_9 | 123.6822 | 137.8416 | 205.8944 |
| hsa-miR-27a-5p | 115.297 | 126.5431 | 41.17888 |
| hsa-miR-505-3p | 115.297 | 137.8416 | 123.5366 |
| novel_84 | 115.297 | 106.2058 | 113.8475 |
| hsa-miR-130b-5p | 115.297 | 65.53124 | 26.64516 |
| hsa-miR-127-5p | 113.2007 | 92.64762 | 101.7361 |
| hsa-miR-362-5p | 111.1044 | 124.2834 | 109.0029 |
| hsa-miR-133a-3p | 109.008 | 90.38792 | 145.3372 |
| hsa-miR-200b-3p | 109.008 | 137.8416 | 38.75659 |
| hsa-miR-339-3p | 109.008 | 108.4655 | 111.4252 |
| hsa-miR-485-5p | 106.9117 | 126.5431 | 96.89148 |
| novel_32 | 104.8154 | 117.5043 | 89.62462 |
| hsa-miR-452-5p | 104.8154 | 103.9461 | 106.5806 |
| hsa-miR-135b-5p | 102.7191 | 72.31034 | 94.46919 |
| hsa-miR-409-5p | 102.7191 | 72.31034 | 75.0909 |
| hsa-miR-29b-1-5p | 102.7191 | 94.90732 | 87.20233 |
| hsa-miR-502-3p | 100.6228 | 103.9461 | 140.4926 |
| novel_44 | 100.6228 | 0 | 48.44574 |
| novel_147 | 100.6228 | 180.7758 | 92.04691 |
| hsa-miR-10a-3p | 98.5265 | 88.12822 | 36.3343 |
| hsa-miR-433-3p | 96.4302 | 124.2834 | 118.6921 |
| hsa-miR-500a-3p | 96.4302 | 119.764 | 118.6921 |
| hsa-miR-485-3p | 96.4302 | 81.34913 | 65.40175 |
| hsa-miR-19a-3p | 94.33389 | 58.75215 | 62.97946 |
| hsa-miR-379-3p | 94.33389 | 97.16702 | 75.0909 |
| hsa-miR-130b-3p | 94.33389 | 54.23275 | 60.55717 |
| hsa-miR-450b-5p | 90.14127 | 58.75215 | 58.13489 |
| hsa-miR-769-5p | 88.04496 | 99.42671 | 87.20233 |
| hsa-miR-144-3p | 88.04496 | 97.16702 | 128.3812 |
| hsa-miR-455-3p | 83.85234 | 92.64762 | 75.0909 |
| hsa-miR-299-5p | 83.85234 | 58.75215 | 70.24632 |
| hsa-miR-708-5p | 81.75604 | 72.31034 | 55.7126 |
| novel_136 | 81.75604 | 92.64762 | 82.35776 |
| hsa-miR-181a-2-3p | 79.65973 | 79.08943 | 96.89148 |
| hsa-miR-487a-3p | 79.65973 | 97.16702 | 82.35776 |
| hsa-miR-296-3p | 77.56342 | 29.37607 | 29.06744 |
| novel_108 | 77.56342 | 40.67457 | 70.24632 |
| hsa-miR-210-3p | 77.56342 | 117.5043 | 169.5601 |
| hsa-miR-424-5p | 75.46711 | 97.16702 | 104.1583 |
| novel_139 | 75.46711 | 0 | 0 |
| novel_68 | 75.46711 | 74.57004 | 62.97946 |
| hsa-miR-4485-3p | 75.46711 | 56.49245 | 109.0029 |
| hsa-miR-29c-3p | 73.3708 | 94.90732 | 65.40175 |
| hsa-miR-151a-5p | 73.3708 | 90.38792 | 89.62462 |
| novel_41 | 73.3708 | 101.6864 | 109.0029 |
| hsa-miR-1296-5p | 73.3708 | 29.37607 | 53.29031 |
| hsa-miR-4326 | 73.3708 | 67.79094 | 46.02345 |
| novel_46 | 71.27449 | 45.19396 | 0 |
| hsa-miR-204-3p | 71.27449 | 106.2058 | 48.44574 |
| hsa-miR-499a-5p | 71.27449 | 88.12822 | 55.7126 |
| novel_110 | 71.27449 | 40.67457 | 118.6921 |
| hsa-miR-500a-5p | 69.17818 | 63.27155 | 75.0909 |
| novel_123 | 69.17818 | 0 | 0 |
| novel_128 | 67.08188 | 42.93426 | 67.82404 |
| novel_10 | 64.98557 | 47.45366 | 60.55717 |
| novel_112 | 64.98557 | 0 | 0 |
| hsa-miR-181a-3p | 64.98557 | 74.57004 | 87.20233 |
| hsa-miR-377-3p | 64.98557 | 47.45366 | 62.97946 |
| novel_63 | 64.98557 | 24.85668 | 65.40175 |
| hsa-miR-532-3p | 62.88926 | 22.59698 | 43.60117 |
| novel_11 | 62.88926 | 67.79094 | 62.97946 |
| novel_135 | 62.88926 | 61.01185 | 55.7126 |
| hsa-miR-331-3p | 60.79295 | 47.45366 | 38.75659 |
| novel_77 | 60.79295 | 126.5431 | 121.1143 |
| hsa-miR-378c | 60.79295 | 47.45366 | 58.13489 |
| hsa-miR-143-5p | 58.69664 | 51.97306 | 50.86803 |
| hsa-miR-582-3p | 58.69664 | 29.37607 | 24.22287 |
| hsa-miR-501-3p | 58.69664 | 36.15517 | 46.02345 |
| hsa-miR-125a-3p | 56.60033 | 33.89547 | 29.06744 |
| hsa-miR-496 | 56.60033 | 38.41487 | 16.95601 |
| novel_53 | 56.60033 | 54.23275 | 150.1818 |
| hsa-miR-708-3p | 56.60033 | 54.23275 | 55.7126 |
| hsa-miR-1-3p | 56.60033 | 38.41487 | 21.80058 |
| hsa-miR-425-3p | 54.50402 | 40.67457 | 62.97946 |
| novel_113 | 54.50402 | 0 | 99.31377 |
| hsa-miR-199b-5p | 54.50402 | 72.31034 | 62.97946 |
| novel_124 | 54.50402 | 0 | 0 |
| hsa-miR-1307-5p | 54.50402 | 38.41487 | 29.06744 |
| hsa-miR-2682-5p | 52.40772 | 38.41487 | 53.29031 |
| hsa-miR-100-3p | 52.40772 | 45.19396 | 41.17888 |
| hsa-miR-539-3p | 50.31141 | 42.93426 | 48.44574 |
| hsa-miR-21-3p | 50.31141 | 40.67457 | 31.48973 |
| hsa-miR-378d | 50.31141 | 49.71336 | 67.82404 |
| hsa-miR-380-3p | 50.31141 | 51.97306 | 53.29031 |
| hsa-let-7a-3p | 50.31141 | 40.67457 | 92.04691 |
| hsa-miR-181d-5p | 48.2151 | 56.49245 | 29.06744 |
| hsa-miR-671-5p | 48.2151 | 29.37607 | 36.3343 |
| hsa-miR-7706 | 48.2151 | 47.45366 | 14.53372 |
| novel_28 | 46.11879 | 79.08943 | 84.78004 |
| hsa-miR-132-5p | 46.11879 | 74.57004 | 65.40175 |
| hsa-miR-15b-3p | 46.11879 | 38.41487 | 16.95601 |
| novel_118 | 46.11879 | 0 | 0 |
| novel_145 | 46.11879 | 0 | 0 |
| novel_125 | 46.11879 | 0 | 0 |
| hsa-miR-32-3p | 46.11879 | 40.67457 | 26.64516 |
| hsa-miR-23a-5p | 46.11879 | 38.41487 | 41.17888 |
| hsa-miR-330-5p | 46.11879 | 67.79094 | 50.86803 |
| hsa-miR-299-3p | 46.11879 | 24.85668 | 36.3343 |
| hsa-miR-501-5p | 46.11879 | 65.53124 | 41.17888 |
| novel_143 | 46.11879 | 0 | 94.46919 |
| hsa-miR-330-3p | 46.11879 | 36.15517 | 55.7126 |
| hsa-miR-576-5p | 44.02248 | 22.59698 | 12.11143 |
| novel_131 | 44.02248 | 0 | 0 |
| novel_78 | 41.92617 | 0 | 0 |
| novel_114 | 41.92617 | 36.15517 | 33.91202 |
| novel_116 | 41.92617 | 0 | 0 |
| novel_40 | 41.92617 | 20.33728 | 33.91202 |
| novel_127 | 41.92617 | 0 | 43.60117 |
| hsa-miR-1228-5p | 41.92617 | 20.33728 | 33.91202 |
| hsa-miR-144-5p | 39.82986 | 67.79094 | 82.35776 |
| novel_95 | 39.82986 | 0 | 0 |
| novel_60 | 39.82986 | 0 | 0 |
| hsa-miR-340-3p | 39.82986 | 29.37607 | 24.22287 |
| hsa-miR-490-3p | 39.82986 | 22.59698 | 24.22287 |
| novel_111 | 39.82986 | 33.89547 | 0 |
| hsa-miR-215-5p | 37.73355 | 42.93426 | 41.17888 |
| novel_29 | 37.73355 | 0 | 0 |
| hsa-miR-431-3p | 37.73355 | 27.11638 | 16.95601 |
| novel_57 | 37.73355 | 31.63577 | 38.75659 |
| novel_101 | 37.73355 | 36.15517 | 31.48973 |
| novel_120 | 37.73355 | 0 | 0 |
| hsa-miR-18a-3p | 35.63725 | 49.71336 | 24.22287 |
| hsa-miR-200c-3p | 35.63725 | 11.29849 | 21.80058 |
| hsa-miR-589-5p | 35.63725 | 24.85668 | 21.80058 |
| hsa-miR-376a-5p | 35.63725 | 27.11638 | 24.22287 |
| hsa-miR-625-3p | 35.63725 | 18.07758 | 12.11143 |
| hsa-miR-584-5p | 35.63725 | 29.37607 | 21.80058 |
| hsa-miR-204-5p | 35.63725 | 42.93426 | 36.3343 |
| novel_12 | 33.54094 | 13.55819 | 0 |
| novel_104 | 33.54094 | 0 | 0 |
| novel_93 | 33.54094 | 0 | 0 |
| hsa-miR-183-5p | 33.54094 | 40.67457 | 24.22287 |
| hsa-miR-4461 | 33.54094 | 18.07758 | 46.02345 |
| hsa-miR-32-5p | 33.54094 | 31.63577 | 48.44574 |
| novel_88 | 33.54094 | 22.59698 | 38.75659 |
| hsa-miR-429 | 31.44463 | 42.93426 | 31.48973 |
| hsa-miR-3180-3p | 31.44463 | 22.59698 | 0 |
| hsa-let-7a-2-3p | 31.44463 | 6.779094 | 9.689148 |
| hsa-miR-1185-2-3p | 31.44463 | 22.59698 | 24.22287 |
| hsa-miR-9-3p | 31.44463 | 13.55819 | 29.06744 |
| novel_27 | 31.44463 | 24.85668 | 33.91202 |
| hsa-miR-3180 | 31.44463 | 22.59698 | 0 |
| novel_82 | 31.44463 | 0 | 43.60117 |
| novel_79 | 29.34832 | 0 | 0 |
| hsa-miR-664a-3p | 29.34832 | 15.81789 | 31.48973 |
| hsa-miR-125b-2-3p | 29.34832 | 27.11638 | 19.3783 |
| novel_19 | 29.34832 | 27.11638 | 0 |
| hsa-miR-550a-3-5p | 29.34832 | 20.33728 | 24.22287 |
| novel_126 | 29.34832 | 58.75215 | 46.02345 |
| hsa-miR-200a-3p | 29.34832 | 36.15517 | 9.689148 |
| hsa-miR-550a-5p | 29.34832 | 18.07758 | 24.22287 |
| novel_13 | 29.34832 | 33.89547 | 19.3783 |
| hsa-miR-10b-3p | 29.34832 | 33.89547 | 31.48973 |
| novel_141 | 29.34832 | 0 | 0 |
| hsa-miR-93-3p | 29.34832 | 13.55819 | 9.689148 |
| hsa-miR-377-5p | 29.34832 | 24.85668 | 29.06744 |
| hsa-miR-598-3p | 29.34832 | 29.37607 | 26.64516 |
| hsa-miR-29c-5p | 27.25201 | 27.11638 | 26.64516 |
| hsa-miR-182-5p | 27.25201 | 36.15517 | 21.80058 |
| hsa-miR-224-3p | 27.25201 | 18.07758 | 4.844574 |
| novel_54 | 27.25201 | 29.37607 | 41.17888 |
| novel_39 | 27.25201 | 0 | 65.40175 |
| hsa-miR-1271-5p | 27.25201 | 18.07758 | 16.95601 |
| hsa-let-7f-1-3p | 27.25201 | 18.07758 | 26.64516 |
| hsa-miR-223-5p | 27.25201 | 29.37607 | 50.86803 |
| novel_156 | 27.25201 | 0 | 0 |
| novel_133 | 27.25201 | 0 | 0 |
| novel_80 | 27.25201 | 0 | 0 |
| novel_33 | 25.1557 | 15.81789 | 19.3783 |
| novel_24 | 25.1557 | 0 | 0 |
| hsa-miR-1301-3p | 25.1557 | 22.59698 | 14.53372 |
| novel_105 | 25.1557 | 0 | 0 |
| novel_148 | 25.1557 | 0 | 31.48973 |
| novel_49 | 25.1557 | 24.85668 | 36.3343 |
| novel_56 | 25.1557 | 22.59698 | 0 |
| novel_67 | 25.1557 | 0 | 19.3783 |
| novel_121 | 25.1557 | 0 | 0 |
| novel_45 | 25.1557 | 0 | 31.48973 |
| hsa-miR-2355-3p | 25.1557 | 20.33728 | 12.11143 |
| novel_146 | 25.1557 | 0 | 0 |
| novel_14 | 25.1557 | 18.07758 | 19.3783 |
| novel_15 | 25.1557 | 24.85668 | 0 |
| novel_99 | 25.1557 | 0 | 0 |
| hsa-miR-129-5p | 25.1557 | 13.55819 | 19.3783 |
| hsa-miR-7-1-3p | 25.1557 | 9.038792 | 16.95601 |
| hsa-miR-1973 | 25.1557 | 15.81789 | 4.844574 |
| hsa-miR-193a-3p | 25.1557 | 29.37607 | 21.80058 |
| hsa-miR-34c-5p | 23.05939 | 24.85668 | 16.95601 |
| hsa-miR-877-5p | 23.05939 | 13.55819 | 21.80058 |
| novel_70 | 23.05939 | 42.93426 | 29.06744 |
| hsa-miR-628-5p | 23.05939 | 11.29849 | 24.22287 |
| hsa-miR-24-1-5p | 23.05939 | 20.33728 | 16.95601 |
| novel_90 | 23.05939 | 0 | 0 |
| hsa-miR-487a-5p | 23.05939 | 29.37607 | 21.80058 |
| hsa-miR-324-5p | 23.05939 | 40.67457 | 31.48973 |
| novel_76 | 23.05939 | 0 | 0 |
| novel_51 | 23.05939 | 0 | 0 |
| hsa-miR-148a-5p | 23.05939 | 31.63577 | 16.95601 |
| novel_31 | 23.05939 | 0 | 0 |
| novel_117 | 20.96309 | 0 | 0 |
| hsa-miR-1287-5p | 20.96309 | 22.59698 | 19.3783 |
| hsa-miR-190a-5p | 20.96309 | 24.85668 | 19.3783 |
| novel_132 | 20.96309 | 0 | 0 |
| hsa-miR-302b-3p | 20.96309 | 18.07758 | 9.689148 |
| novel_50 | 20.96309 | 9.038792 | 7.266861 |
| novel_92 | 20.96309 | 0 | 41.17888 |
| novel_152 | 20.96309 | 0 | 0 |
| hsa-miR-191-3p | 20.96309 | 24.85668 | 0 |
| hsa-miR-887-3p | 18.86678 | 9.038792 | 7.266861 |
| novel_96 | 18.86678 | 27.11638 | 0 |
| hsa-miR-203a-3p | 18.86678 | 11.29849 | 14.53372 |
| hsa-miR-491-5p | 18.86678 | 6.779094 | 9.689148 |
| hsa-miR-27b-5p | 18.86678 | 22.59698 | 4.844574 |
| novel_153 | 18.86678 | 0 | 0 |
| hsa-miR-338-3p | 18.86678 | 6.779094 | 12.11143 |
| hsa-miR-940 | 18.86678 | 9.038792 | 4.844574 |
| hsa-miR-195-5p | 18.86678 | 18.07758 | 19.3783 |
| novel_16 | 18.86678 | 18.07758 | 0 |
| hsa-miR-9-5p | 18.86678 | 6.779094 | 50.86803 |
| novel_130 | 18.86678 | 0 | 9.689148 |
| hsa-miR-375 | 18.86678 | 2.259698 | 4.844574 |
| novel_119 | 18.86678 | 0 | 0 |
| hsa-miR-3909 | 18.86678 | 15.81789 | 9.689148 |
| hsa-miR-296-5p | 18.86678 | 13.55819 | 12.11143 |
| hsa-miR-1306-5p | 16.77047 | 9.038792 | 2.422287 |
| hsa-miR-374a-3p | 16.77047 | 18.07758 | 4.844574 |
| novel_142 | 16.77047 | 0 | 0 |
| hsa-miR-4775 | 16.77047 | 20.33728 | 14.53372 |
| hsa-miR-365b-5p | 16.77047 | 6.779094 | 19.3783 |
| hsa-miR-1260b | 16.77047 | 20.33728 | 9.689148 |
| hsa-miR-30c-2-3p | 16.77047 | 15.81789 | 26.64516 |
| hsa-miR-380-5p | 16.77047 | 15.81789 | 4.844574 |
| hsa-miR-873-5p | 16.77047 | 6.779094 | 4.844574 |
| hsa-miR-615-5p | 16.77047 | 2.259698 | 16.95601 |
| hsa-miR-542-3p | 16.77047 | 18.07758 | 14.53372 |
| hsa-miR-6724-5p | 16.77047 | 27.11638 | 0 |
| hsa-miR-624-5p | 16.77047 | 2.259698 | 2.422287 |
| novel_17 | 16.77047 | 0 | 26.64516 |
| novel_155 | 14.67416 | 0 | 0 |
| hsa-miR-378a-5p | 14.67416 | 11.29849 | 14.53372 |
| novel_134 | 14.67416 | 0 | 0 |
| hsa-miR-26b-3p | 14.67416 | 4.519396 | 0 |
| hsa-miR-2110 | 14.67416 | 4.519396 | 19.3783 |
| hsa-miR-362-3p | 14.67416 | 24.85668 | 9.689148 |
| novel_154 | 14.67416 | 24.85668 | 0 |
| novel_140 | 14.67416 | 0 | 14.53372 |
| hsa-miR-760 | 14.67416 | 13.55819 | 7.266861 |
| hsa-miR-323a-5p | 14.67416 | 15.81789 | 14.53372 |
| hsa-miR-205-5p | 14.67416 | 38.41487 | 16.95601 |
| hsa-miR-324-3p | 14.67416 | 9.038792 | 4.844574 |
| hsa-miR-188-5p | 14.67416 | 20.33728 | 31.48973 |
| hsa-miR-181c-5p | 14.67416 | 11.29849 | 12.11143 |
| novel_149 | 14.67416 | 0 | 0 |
| hsa-miR-629-3p | 14.67416 | 2.259698 | 7.266861 |
| hsa-miR-494-5p | 14.67416 | 2.259698 | 2.422287 |
| hsa-miR-505-5p | 14.67416 | 11.29849 | 9.689148 |
| novel_151 | 14.67416 | 0 | 0 |
| novel_18 | 14.67416 | 0 | 0 |
| hsa-miR-138-5p | 14.67416 | 11.29849 | 9.689148 |
| hsa-miR-193b-5p | 12.57785 | 18.07758 | 21.80058 |
| hsa-miR-4677-3p | 12.57785 | 11.29849 | 7.266861 |
| hsa-miR-1197 | 12.57785 | 11.29849 | 4.844574 |
| hsa-miR-548o-3p | 12.57785 | 27.11638 | 43.60117 |
| hsa-miR-1908-5p | 12.57785 | 4.519396 | 7.266861 |
| hsa-miR-500b-5p | 12.57785 | 9.038792 | 21.80058 |
| hsa-miR-301a-3p | 12.57785 | 9.038792 | 4.844574 |
| hsa-miR-3130-3p | 12.57785 | 4.519396 | 4.844574 |
| hsa-miR-618 | 12.57785 | 6.779094 | 9.689148 |
| hsa-miR-4466 | 12.57785 | 0 | 4.844574 |
| hsa-miR-4508 | 12.57785 | 2.259698 | 2.422287 |
| hsa-miR-628-3p | 12.57785 | 22.59698 | 12.11143 |
| hsa-miR-342-5p | 12.57785 | 11.29849 | 4.844574 |
| hsa-miR-3158-3p | 12.57785 | 4.519396 | 19.3783 |
| hsa-miR-1226-5p | 12.57785 | 6.779094 | 0 |
| hsa-miR-326 | 12.57785 | 11.29849 | 19.3783 |
| hsa-miR-412-3p | 12.57785 | 13.55819 | 7.266861 |
| hsa-miR-17-3p | 10.48154 | 6.779094 | 7.266861 |
| hsa-miR-3117-3p | 10.48154 | 0 | 2.422287 |
| hsa-miR-665 | 10.48154 | 2.259698 | 4.844574 |
| hsa-miR-4677-5p | 10.48154 | 0 | 0 |
| hsa-let-7e-3p | 10.48154 | 6.779094 | 9.689148 |
| hsa-miR-664a-5p | 10.48154 | 4.519396 | 12.11143 |
| hsa-miR-365a-5p | 10.48154 | 6.779094 | 2.422287 |
| hsa-miR-185-3p | 10.48154 | 0 | 0 |
| hsa-miR-769-3p | 10.48154 | 6.779094 | 16.95601 |
| hsa-miR-668-3p | 10.48154 | 9.038792 | 9.689148 |
| hsa-miR-541-5p | 10.48154 | 33.89547 | 16.95601 |
| hsa-miR-374b-3p | 10.48154 | 4.519396 | 4.844574 |
| hsa-miR-3591-5p | 10.48154 | 9.038792 | 7.266861 |
| hsa-miR-383-5p | 10.48154 | 4.519396 | 2.422287 |
| hsa-miR-1306-3p | 10.48154 | 13.55819 | 14.53372 |
| hsa-miR-1292-5p | 10.48154 | 2.259698 | 2.422287 |
| hsa-miR-4792 | 10.48154 | 0 | 2.422287 |
| hsa-miR-454-5p | 10.48154 | 2.259698 | 2.422287 |
| hsa-miR-96-5p | 10.48154 | 6.779094 | 0 |
| hsa-miR-6511b-3p | 8.385234 | 4.519396 | 4.844574 |
| hsa-miR-376a-2-5p | 8.385234 | 4.519396 | 2.422287 |
| hsa-miR-502-5p | 8.385234 | 9.038792 | 4.844574 |
| hsa-miR-4463 | 8.385234 | 0 | 12.11143 |
| hsa-miR-92a-1-5p | 8.385234 | 4.519396 | 12.11143 |
| hsa-miR-208b-3p | 8.385234 | 20.33728 | 9.689148 |
| hsa-miR-5010-3p | 8.385234 | 4.519396 | 2.422287 |
| hsa-miR-935 | 8.385234 | 13.55819 | 16.95601 |
| hsa-miR-26a-2-3p | 8.385234 | 22.59698 | 0 |
| hsa-miR-3129-5p | 8.385234 | 2.259698 | 0 |
| hsa-miR-548i | 8.385234 | 9.038792 | 0 |
| hsa-miR-3129-3p | 8.385234 | 6.779094 | 2.422287 |
| hsa-miR-212-3p | 8.385234 | 11.29849 | 9.689148 |
| hsa-miR-34c-3p | 8.385234 | 13.55819 | 7.266861 |
| hsa-miR-376c-5p | 8.385234 | 13.55819 | 19.3783 |
| hsa-miR-135a-5p | 8.385234 | 13.55819 | 4.844574 |
| hsa-miR-6511a-3p | 8.385234 | 0 | 0 |
| hsa-miR-4485-5p | 8.385234 | 2.259698 | 12.11143 |
| hsa-miR-3179 | 8.385234 | 9.038792 | 0 |
| hsa-miR-33a-5p | 8.385234 | 18.07758 | 7.266861 |
| novel_20 | 8.385234 | 0 | 9.689148 |
| hsa-miR-216b-5p | 8.385234 | 0 | 2.422287 |
| hsa-miR-486-3p | 8.385234 | 0 | 0 |
| hsa-miR-1910-5p | 6.288926 | 0 | 0 |
| novel_72 | 6.288926 | 0 | 0 |
| hsa-miR-133b | 6.288926 | 9.038792 | 7.266861 |
| hsa-miR-200b-5p | 6.288926 | 0 | 2.422287 |
| hsa-miR-152-5p | 6.288926 | 11.29849 | 2.422287 |
| hsa-miR-616-5p | 6.288926 | 4.519396 | 0 |
| hsa-miR-2355-5p | 6.288926 | 6.779094 | 2.422287 |
| hsa-miR-582-5p | 6.288926 | 15.81789 | 9.689148 |
| hsa-miR-766-3p | 6.288926 | 13.55819 | 7.266861 |
| hsa-miR-370-5p | 6.288926 | 11.29849 | 2.422287 |
| hsa-miR-1291 | 6.288926 | 11.29849 | 4.844574 |
| hsa-miR-550a-3p | 6.288926 | 0 | 0 |
| hsa-miR-3940-3p | 6.288926 | 0 | 4.844574 |
| hsa-miR-219a-5p | 6.288926 | 0 | 0 |
| hsa-miR-148b-5p | 6.288926 | 13.55819 | 4.844574 |
| hsa-miR-1284 | 6.288926 | 0 | 2.422287 |
| hsa-miR-147b | 6.288926 | 33.89547 | 14.53372 |
| hsa-let-7i-3p | 6.288926 | 9.038792 | 4.844574 |
| hsa-miR-3152-5p | 6.288926 | 13.55819 | 14.53372 |
| hsa-miR-1910-3p | 6.288926 | 6.779094 | 2.422287 |
| hsa-miR-1248 | 6.288926 | 9.038792 | 2.422287 |
| hsa-miR-1246 | 6.288926 | 9.038792 | 2.422287 |
| hsa-miR-4516 | 6.288926 | 2.259698 | 7.266861 |
| hsa-miR-2276-3p | 6.288926 | 18.07758 | 4.844574 |
| hsa-miR-642a-3p | 6.288926 | 0 | 0 |
| hsa-miR-651-5p | 6.288926 | 4.519396 | 12.11143 |
| hsa-miR-660-3p | 6.288926 | 13.55819 | 12.11143 |
| hsa-miR-301a-5p | 6.288926 | 6.779094 | 0 |
| hsa-miR-671-3p | 6.288926 | 9.038792 | 7.266861 |
| hsa-miR-122-3p | 6.288926 | 6.779094 | 9.689148 |
| hsa-miR-3617-5p | 6.288926 | 6.779094 | 7.266861 |
| hsa-miR-1293 | 6.288926 | 0 | 4.844574 |
| hsa-miR-1228-3p | 6.288926 | 0 | 0 |
| hsa-miR-6770-3p | 6.288926 | 6.779094 | 0 |
| hsa-miR-302d-3p | 6.288926 | 4.519396 | 4.844574 |
| hsa-miR-483-3p | 6.288926 | 13.55819 | 24.22287 |
| hsa-miR-1294 | 6.288926 | 0 | 0 |
| hsa-miR-106a-5p | 6.288926 | 11.29849 | 4.844574 |
| hsa-miR-33b-5p | 6.288926 | 2.259698 | 0 |
| hsa-miR-7108-5p | 6.288926 | 4.519396 | 0 |
| hsa-miR-34b-3p | 6.288926 | 2.259698 | 4.844574 |
| hsa-miR-641 | 6.288926 | 6.779094 | 4.844574 |
| hsa-miR-25-5p | 6.288926 | 2.259698 | 0 |
| novel_21 | 6.288926 | 0 | 2.422287 |
| hsa-miR-181c-3p | 4.192617 | 13.55819 | 2.422287 |
| hsa-miR-770-5p | 4.192617 | 6.779094 | 7.266861 |
| hsa-miR-942-5p | 4.192617 | 15.81789 | 2.422287 |
| novel_22 | 4.192617 | 0 | 0 |
| hsa-miR-29b-2-5p | 4.192617 | 9.038792 | 7.266861 |
| hsa-miR-95-3p | 4.192617 | 6.779094 | 2.422287 |
| hsa-miR-141-3p | 4.192617 | 0 | 2.422287 |
| hsa-miR-664b-3p | 4.192617 | 4.519396 | 2.422287 |
| novel_26 | 4.192617 | 0 | 0 |
| novel_75 | 4.192617 | 0 | 9.689148 |
| hsa-miR-3679-5p | 4.192617 | 0 | 7.266861 |
| hsa-miR-329-5p | 4.192617 | 0 | 4.844574 |
| hsa-miR-4721 | 4.192617 | 0 | 0 |
| hsa-miR-1249-3p | 4.192617 | 6.779094 | 9.689148 |
| hsa-miR-497-5p | 4.192617 | 0 | 2.422287 |
| hsa-miR-3200-3p | 4.192617 | 2.259698 | 0 |
| hsa-miR-2116-3p | 4.192617 | 0 | 0 |
| hsa-miR-3115 | 4.192617 | 4.519396 | 0 |
| hsa-miR-6516-5p | 4.192617 | 4.519396 | 0 |
| hsa-miR-548k | 4.192617 | 0 | 0 |
| hsa-miR-338-5p | 4.192617 | 6.779094 | 2.422287 |
| hsa-miR-483-5p | 4.192617 | 9.038792 | 2.422287 |
| hsa-miR-30c-1-3p | 4.192617 | 0 | 2.422287 |
| hsa-miR-3613-3p | 4.192617 | 0 | 2.422287 |
| hsa-miR-376b-5p | 4.192617 | 15.81789 | 16.95601 |
| hsa-miR-3074-5p | 4.192617 | 0 | 0 |
| hsa-miR-1278 | 4.192617 | 4.519396 | 2.422287 |
| hsa-miR-302a-3p | 4.192617 | 9.038792 | 7.266861 |
| hsa-miR-541-3p | 4.192617 | 2.259698 | 2.422287 |
| hsa-miR-153-3p | 4.192617 | 4.519396 | 4.844574 |
| novel_23 | 4.192617 | 0 | 14.53372 |
| hsa-miR-6723-5p | 4.192617 | 0 | 2.422287 |
| hsa-miR-6511b-5p | 4.192617 | 4.519396 | 0 |
| hsa-miR-134-3p | 4.192617 | 4.519396 | 0 |
| hsa-miR-1245b-3p | 4.192617 | 0 | 2.422287 |
| hsa-miR-655-5p | 4.192617 | 2.259698 | 0 |
| hsa-miR-376b-3p | 4.192617 | 11.29849 | 12.11143 |
| hsa-miR-206 | 4.192617 | 4.519396 | 4.844574 |
| hsa-miR-1185-5p | 4.192617 | 9.038792 | 0 |
| hsa-miR-3177-3p | 4.192617 | 4.519396 | 2.422287 |
| hsa-miR-487b-5p | 4.192617 | 2.259698 | 4.844574 |
| hsa-miR-3934-5p | 4.192617 | 9.038792 | 2.422287 |
| hsa-miR-433-5p | 4.192617 | 2.259698 | 0 |
| hsa-miR-4725-3p | 4.192617 | 4.519396 | 2.422287 |
| hsa-miR-302a-5p | 4.192617 | 0 | 0 |
| hsa-miR-30b-3p | 4.192617 | 4.519396 | 2.422287 |
| hsa-miR-1267 | 4.192617 | 4.519396 | 2.422287 |
| hsa-miR-33b-3p | 4.192617 | 2.259698 | 7.266861 |
| hsa-miR-6769b-3p | 4.192617 | 0 | 0 |
| hsa-miR-19b-1-5p | 4.192617 | 2.259698 | 2.422287 |
| hsa-miR-542-5p | 4.192617 | 11.29849 | 9.689148 |
| hsa-miR-548e-3p | 4.192617 | 2.259698 | 0 |
| hsa-miR-597-3p | 4.192617 | 6.779094 | 4.844574 |
| hsa-miR-590-5p | 4.192617 | 0 | 2.422287 |
| hsa-miR-4685-3p | 4.192617 | 2.259698 | 0 |
| hsa-miR-105-5p | 4.192617 | 4.519396 | 4.844574 |
| hsa-let-7b-3p | 4.192617 | 13.55819 | 4.844574 |
| hsa-miR-3648 | 4.192617 | 9.038792 | 4.844574 |
| hsa-miR-885-5p | 4.192617 | 6.779094 | 7.266861 |
| hsa-miR-1276 | 4.192617 | 4.519396 | 0 |
| hsa-miR-23b-5p | 4.192617 | 6.779094 | 7.266861 |
| hsa-miR-1277-5p | 4.192617 | 2.259698 | 0 |
| hsa-miR-3150a-5p | 4.192617 | 0 | 0 |
| hsa-miR-1179 | 2.096309 | 4.519396 | 2.422287 |
| hsa-miR-4457 | 2.096309 | 0 | 0 |
| hsa-miR-4467 | 2.096309 | 0 | 2.422287 |
| hsa-miR-2277-3p | 2.096309 | 0 | 2.422287 |
| hsa-miR-556-5p | 2.096309 | 0 | 2.422287 |
| hsa-miR-98-3p | 2.096309 | 6.779094 | 4.844574 |
| hsa-miR-99a-3p | 2.096309 | 4.519396 | 2.422287 |
| hsa-miR-3929 | 2.096309 | 0 | 0 |
| hsa-miR-2114-3p | 2.096309 | 0 | 2.422287 |
| novel_34 | 2.096309 | 0 | 0 |
| hsa-miR-301b-3p | 2.096309 | 0 | 9.689148 |
| hsa-miR-4788 | 2.096309 | 0 | 0 |
| hsa-miR-4504 | 2.096309 | 0 | 0 |
| hsa-miR-511-5p | 2.096309 | 0 | 0 |
| hsa-miR-6741-5p | 2.096309 | 0 | 0 |
| hsa-miR-4661-5p | 2.096309 | 0 | 0 |
| hsa-miR-4659a-3p | 2.096309 | 0 | 0 |
| hsa-miR-6828-3p | 2.096309 | 0 | 0 |
| hsa-miR-1229-3p | 2.096309 | 0 | 2.422287 |
| hsa-miR-5088-5p | 2.096309 | 0 | 0 |
| hsa-miR-128-1-5p | 2.096309 | 2.259698 | 4.844574 |
| hsa-miR-3176 | 2.096309 | 0 | 2.422287 |
| hsa-miR-6850-5p | 2.096309 | 0 | 0 |
| hsa-miR-642a-5p | 2.096309 | 4.519396 | 0 |
| hsa-miR-3148 | 2.096309 | 0 | 2.422287 |
| hsa-miR-4688 | 2.096309 | 0 | 0 |
| hsa-miR-20b-5p | 2.096309 | 0 | 0 |
| hsa-miR-196b-3p | 2.096309 | 0 | 0 |
| hsa-miR-585-3p | 2.096309 | 0 | 0 |
| hsa-miR-4709-5p | 2.096309 | 0 | 0 |
| hsa-miR-3143 | 2.096309 | 2.259698 | 0 |
| hsa-miR-92b-5p | 2.096309 | 6.779094 | 9.689148 |
| hsa-miR-18b-5p | 2.096309 | 0 | 2.422287 |
| hsa-miR-5706 | 2.096309 | 0 | 0 |
| hsa-miR-3173-5p | 2.096309 | 0 | 0 |
| hsa-miR-544a | 2.096309 | 4.519396 | 2.422287 |
| hsa-miR-302c-3p | 2.096309 | 4.519396 | 4.844574 |
| hsa-miR-4660 | 2.096309 | 0 | 0 |
| hsa-miR-939-5p | 2.096309 | 0 | 2.422287 |
| hsa-miR-549a | 2.096309 | 2.259698 | 0 |
| hsa-miR-548p | 2.096309 | 0 | 0 |
| hsa-miR-1288-3p | 2.096309 | 2.259698 | 4.844574 |
| hsa-miR-3681-5p | 2.096309 | 2.259698 | 0 |
| hsa-miR-3065-5p | 2.096309 | 4.519396 | 0 |
| hsa-miR-7976 | 2.096309 | 0 | 0 |
| hsa-miR-190b | 2.096309 | 4.519396 | 4.844574 |
| hsa-miR-1193 | 2.096309 | 0 | 0 |
| hsa-miR-3942-3p | 2.096309 | 0 | 0 |
| hsa-miR-659-5p | 2.096309 | 2.259698 | 4.844574 |
| hsa-miR-6790-5p | 2.096309 | 0 | 0 |
| hsa-miR-6885-5p | 2.096309 | 0 | 0 |
| hsa-miR-1262 | 2.096309 | 0 | 0 |
| hsa-miR-6754-3p | 2.096309 | 0 | 0 |
| hsa-miR-876-3p | 2.096309 | 4.519396 | 0 |
| hsa-miR-1909-5p | 2.096309 | 0 | 0 |
| hsa-miR-181b-3p | 2.096309 | 0 | 0 |
| hsa-miR-6804-5p | 2.096309 | 2.259698 | 0 |
| hsa-miR-518f-3p | 2.096309 | 0 | 0 |
| hsa-miR-3605-3p | 2.096309 | 0 | 2.422287 |
| hsa-miR-605-3p | 2.096309 | 0 | 2.422287 |
| hsa-miR-548j-5p | 2.096309 | 4.519396 | 0 |
| hsa-miR-219a-1-3p | 2.096309 | 0 | 2.422287 |
| hsa-miR-195-3p | 2.096309 | 0 | 7.266861 |
| hsa-miR-33a-3p | 2.096309 | 2.259698 | 0 |
| hsa-miR-4676-5p | 2.096309 | 2.259698 | 2.422287 |
| hsa-miR-5009-5p | 2.096309 | 0 | 0 |
| hsa-miR-363-3p | 2.096309 | 2.259698 | 9.689148 |
| hsa-miR-4755-3p | 2.096309 | 0 | 0 |
| hsa-let-7g-3p | 2.096309 | 0 | 0 |
| hsa-miR-495-5p | 2.096309 | 11.29849 | 2.422287 |
| hsa-miR-6736-5p | 2.096309 | 0 | 0 |
| hsa-miR-410-5p | 2.096309 | 0 | 0 |
| hsa-miR-767-5p | 2.096309 | 2.259698 | 0 |
| hsa-miR-3614-5p | 2.096309 | 0 | 0 |
| hsa-miR-6869-5p | 2.096309 | 0 | 0 |
| hsa-miR-202-5p | 2.096309 | 6.779094 | 2.422287 |
| hsa-miR-5001-3p | 2.096309 | 0 | 2.422287 |
| hsa-miR-4449 | 2.096309 | 0 | 2.422287 |
| hsa-miR-6821-5p | 2.096309 | 4.519396 | 0 |
| hsa-miR-6866-5p | 2.096309 | 2.259698 | 2.422287 |
| hsa-miR-3187-3p | 2.096309 | 2.259698 | 0 |
| hsa-miR-4636 | 2.096309 | 2.259698 | 2.422287 |
| hsa-miR-643 | 2.096309 | 0 | 0 |
| hsa-miR-3675-5p | 2.096309 | 0 | 4.844574 |
| hsa-miR-184 | 2.096309 | 0 | 7.266861 |
| hsa-miR-4423-5p | 2.096309 | 0 | 0 |
| hsa-miR-3661 | 2.096309 | 0 | 0 |
| hsa-miR-6842-3p | 2.096309 | 2.259698 | 0 |
| hsa-miR-4664-3p | 2.096309 | 0 | 0 |
| hsa-miR-196a-3p | 2.096309 | 0 | 0 |
| hsa-miR-942-3p | 2.096309 | 2.259698 | 0 |
| hsa-miR-889-5p | 2.096309 | 2.259698 | 0 |
| hsa-miR-449a | 2.096309 | 2.259698 | 2.422287 |
| hsa-let-7c-3p | 2.096309 | 0 | 0 |
| hsa-miR-5579-5p | 2.096309 | 0 | 2.422287 |
| hsa-miR-6858-5p | 2.096309 | 4.519396 | 0 |
| hsa-miR-4741 | 2.096309 | 0 | 0 |
| hsa-miR-5000-3p | 2.096309 | 0 | 0 |
| hsa-miR-6735-5p | 2.096309 | 0 | 0 |
| hsa-miR-4762-3p | 2.096309 | 0 | 0 |
| hsa-miR-873-3p | 2.096309 | 6.779094 | 2.422287 |
| hsa-miR-6819-5p | 2.096309 | 0 | 0 |
| hsa-miR-450a-1-3p | 2.096309 | 2.259698 | 0 |
| hsa-miR-6889-5p | 2.096309 | 0 | 0 |
| hsa-miR-5582-3p | 2.096309 | 4.519396 | 4.844574 |
| hsa-miR-4704-5p | 2.096309 | 0 | 0 |
| hsa-miR-6818-3p | 2.096309 | 2.259698 | 0 |
| hsa-miR-6891-5p | 2.096309 | 2.259698 | 0 |
| hsa-let-7f-2-3p | 2.096309 | 0 | 4.844574 |
| hsa-miR-8072 | 2.096309 | 0 | 0 |
| hsa-miR-5100 | 2.096309 | 0 | 0 |
| hsa-miR-1277-3p | 2.096309 | 9.038792 | 12.11143 |
| hsa-miR-6721-5p | 2.096309 | 0 | 0 |
| hsa-miR-579-3p | 2.096309 | 2.259698 | 4.844574 |
| hsa-miR-1468-5p | 2.096309 | 0 | 0 |
| hsa-miR-3677-3p | 2.096309 | 0 | 0 |
| hsa-miR-4684-3p | 2.096309 | 0 | 0 |
| hsa-miR-6516-3p | 2.096309 | 0 | 0 |
| hsa-miR-663a | 2.096309 | 2.259698 | 0 |
| hsa-miR-545-5p | 2.096309 | 2.259698 | 0 |
| hsa-miR-187-3p | 2.096309 | 0 | 0 |
| hsa-miR-6783-5p | 2.096309 | 0 | 0 |
| hsa-miR-1180-5p | 2.096309 | 0 | 0 |
| hsa-miR-4725-5p | 2.096309 | 0 | 0 |
| hsa-miR-627-3p | 2.096309 | 9.038792 | 2.422287 |
| hsa-miR-2682-3p | 2.096309 | 6.779094 | 9.689148 |
| novel_174 | 0 | 2926.309 | 0 |
| hsa-miR-301b-5p | 0 | 2.259698 | 0 |
| novel_173 | 0 | 92.64762 | 0 |
| novel_177 | 0 | 38.41487 | 0 |
| hsa-miR-943 | 0 | 2.259698 | 0 |
| novel_179 | 0 | 6.779094 | 0 |
| hsa-miR-124-3p | 0 | 0 | 7.266861 |
| novel_178 | 0 | 49.71336 | 0 |
| novel_192 | 0 | 736.6616 | 0 |
| novel_175 | 0 | 61.01185 | 0 |
| novel_195 | 0 | 0 | 9.689148 |
| hsa-miR-3126-3p | 0 | 0 | 2.422287 |
| hsa-miR-3125 | 0 | 0 | 4.844574 |
| novel_191 | 0 | 13.55819 | 0 |
| hsa-miR-210-5p | 0 | 0 | 2.422287 |
| hsa-miR-381-5p | 0 | 2.259698 | 0 |
| novel_187 | 0 | 31.63577 | 0 |
| novel_223 | 0 | 0 | 16.95601 |
| hsa-miR-6877-5p | 0 | 2.259698 | 0 |
| hsa-miR-6745 | 0 | 2.259698 | 0 |
| hsa-miR-4766-3p | 0 | 6.779094 | 2.422287 |
| novel_185 | 0 | 1873.29 | 0 |
| novel_184 | 0 | 2225.803 | 0 |
| hsa-miR-4676-3p | 0 | 0 | 2.422287 |
| novel_182 | 0 | 47.45366 | 0 |
| novel_181 | 0 | 1337.741 | 0 |
| hsa-miR-1538 | 0 | 2.259698 | 4.844574 |
| novel_172 | 0 | 664.3512 | 0 |
| hsa-miR-2277-5p | 0 | 2.259698 | 2.422287 |
| hsa-miR-6837-3p | 0 | 2.259698 | 0 |
| hsa-miR-1305 | 0 | 2.259698 | 4.844574 |
| novel_170 | 0 | 29.37607 | 0 |
| hsa-miR-5682 | 0 | 2.259698 | 0 |
| novel_206 | 0 | 0 | 36.3343 |
| hsa-miR-744-3p | 0 | 4.519396 | 2.422287 |
| novel_194 | 0 | 0 | 16.95601 |
| novel_164 | 0 | 11.29849 | 0 |
| novel_163 | 0 | 20.33728 | 0 |
| novel_224 | 0 | 0 | 147.7595 |
| novel_162 | 0 | 4.519396 | 0 |
| novel_210 | 0 | 0 | 26.64516 |
| novel_211 | 0 | 0 | 43.60117 |
| novel_212 | 0 | 0 | 26.64516 |
| novel_213 | 0 | 0 | 479.6128 |
| novel_176 | 0 | 664.3512 | 0 |
| novel_214 | 0 | 0 | 24.22287 |
| novel_215 | 0 | 0 | 8528.872 |
| novel_216 | 0 | 0 | 21.80058 |
| novel_217 | 0 | 0 | 31.48973 |
| novel_218 | 0 | 0 | 169.5601 |
| novel_219 | 0 | 0 | 26.64516 |
| hsa-miR-323b-5p | 0 | 2.259698 | 0 |
| novel_220 | 0 | 0 | 19.3783 |
| novel_221 | 0 | 0 | 8722.655 |
| novel_222 | 0 | 0 | 419.0556 |
| novel_207 | 0 | 0 | 46.02345 |
| novel_205 | 0 | 0 | 41.17888 |
| hsa-miR-1231 | 0 | 2.259698 | 0 |
| novel_204 | 0 | 0 | 75.0909 |
| hsa-miR-625-5p | 0 | 4.519396 | 14.53372 |
| novel_171 | 0 | 85.86853 | 0 |
| novel_193 | 0 | 90.38792 | 0 |
| novel_159 | 0 | 11.29849 | 0 |
| novel_161 | 0 | 11.29849 | 0 |
| novel_160 | 0 | 9.038792 | 0 |
| novel_208 | 0 | 0 | 87.20233 |
| novel_196 | 0 | 0 | 36.3343 |
| novel_197 | 0 | 0 | 343.9648 |
| novel_168 | 0 | 951.3329 | 0 |
| novel_198 | 0 | 0 | 118.6921 |
| novel_199 | 0 | 0 | 48.44574 |
| novel_225 | 0 | 0 | 31.48973 |
| novel_167 | 0 | 2.259698 | 0 |
| novel_166 | 0 | 31.63577 | 0 |
| hsa-miR-3065-3p | 0 | 0 | 2.422287 |
| novel_200 | 0 | 0 | 40435.24 |
| novel_201 | 0 | 0 | 314.8973 |
| novel_202 | 0 | 0 | 87.20233 |
| novel_165 | 0 | 33.89547 | 0 |
| novel_203 | 0 | 0 | 16.95601 |
| novel_209 | 0 | 0 | 26.64516 |
| hsa-miR-6754-5p | 0 | 0 | 2.422287 |
| hsa-miR-1908-3p | 0 | 2.259698 | 0 |
| hsa-miR-1254 | 0 | 0 | 4.844574 |
| hsa-miR-6767-5p | 0 | 0 | 2.422287 |
| hsa-miR-1245a | 0 | 2.259698 | 2.422287 |
| hsa-miR-3124-5p | 0 | 2.259698 | 0 |
| hsa-miR-597-5p | 0 | 2.259698 | 0 |
| hsa-miR-584-3p | 0 | 0 | 2.422287 |
| hsa-miR-129-1-3p | 0 | 2.259698 | 0 |
| hsa-miR-4668-5p | 0 | 2.259698 | 2.422287 |
| hsa-miR-200a-5p | 0 | 6.779094 | 0 |
| hsa-miR-1915-3p | 0 | 0 | 2.422287 |
| hsa-miR-1252-5p | 0 | 2.259698 | 0 |
| hsa-miR-212-5p | 0 | 2.259698 | 0 |
| hsa-miR-3605-5p | 0 | 4.519396 | 4.844574 |
| hsa-miR-450a-2-3p | 0 | 4.519396 | 2.422287 |
| hsa-miR-6747-3p | 0 | 2.259698 | 0 |
| hsa-miR-208a-3p | 0 | 0 | 2.422287 |
| hsa-miR-6764-5p | 0 | 0 | 2.422287 |
| hsa-miR-374c-5p | 0 | 0 | 2.422287 |
| hsa-miR-4473 | 0 | 4.519396 | 4.844574 |
| hsa-miR-3126-5p | 0 | 0 | 2.422287 |
| hsa-miR-3685 | 0 | 2.259698 | 0 |
| hsa-miR-3687 | 0 | 4.519396 | 0 |
| hsa-miR-139-3p | 0 | 2.259698 | 7.266861 |
| hsa-miR-7156-3p | 0 | 2.259698 | 0 |
| hsa-miR-4781-3p | 0 | 2.259698 | 0 |
| hsa-miR-3138 | 0 | 2.259698 | 2.422287 |
| hsa-miR-6515-5p | 0 | 2.259698 | 0 |
| hsa-miR-937-3p | 0 | 4.519396 | 0 |
| hsa-miR-7641 | 0 | 0 | 4.844574 |
| hsa-miR-548q | 0 | 2.259698 | 0 |
| hsa-miR-548u | 0 | 2.259698 | 0 |
| hsa-miR-6875-5p | 0 | 0 | 2.422287 |
| hsa-miR-3918 | 0 | 2.259698 | 0 |
| hsa-miR-1255a | 0 | 2.259698 | 0 |
| hsa-miR-548l | 0 | 2.259698 | 2.422287 |
| hsa-miR-503-3p | 0 | 0 | 2.422287 |
| hsa-miR-874-5p | 0 | 0 | 2.422287 |
| hsa-miR-6779-5p | 0 | 2.259698 | 0 |
| hsa-miR-6883-3p | 0 | 0 | 2.422287 |
| hsa-miR-5699-3p | 0 | 2.259698 | 0 |
| hsa-miR-6852-5p | 0 | 0 | 9.689148 |
| hsa-miR-659-3p | 0 | 0 | 2.422287 |
| hsa-miR-129-2-3p | 0 | 0 | 4.844574 |
| hsa-miR-4707-3p | 0 | 2.259698 | 0 |
| hsa-miR-520g-3p | 0 | 0 | 2.422287 |
| hsa-miR-3912-3p | 0 | 2.259698 | 2.422287 |
| hsa-miR-6732-5p | 0 | 0 | 2.422287 |
| hsa-miR-4665-5p | 0 | 0 | 2.422287 |
| hsa-miR-6828-5p | 0 | 0 | 2.422287 |
| hsa-miR-656-5p | 0 | 2.259698 | 0 |
| hsa-miR-6757-5p | 0 | 4.519396 | 0 |
| hsa-miR-5581-3p | 0 | 2.259698 | 0 |
| hsa-miR-151b | 0 | 9.038792 | 2.422287 |
| hsa-miR-6784-3p | 0 | 0 | 2.422287 |
| hsa-miR-449c-5p | 0 | 0 | 2.422287 |
| hsa-miR-6716-3p | 0 | 4.519396 | 0 |
| hsa-miR-4425 | 0 | 0 | 2.422287 |
| hsa-miR-3199 | 0 | 4.519396 | 9.689148 |
| hsa-miR-320e | 0 | 0 | 2.422287 |
| hsa-miR-6799-3p | 0 | 2.259698 | 0 |
| hsa-miR-2278 | 0 | 0 | 4.844574 |
| hsa-miR-1322 | 0 | 2.259698 | 0 |
| hsa-miR-605-5p | 0 | 2.259698 | 0 |
| hsa-miR-3164 | 0 | 2.259698 | 0 |
| hsa-miR-34b-5p | 0 | 0 | 2.422287 |
| hsa-miR-1343-3p | 0 | 11.29849 | 12.11143 |
| hsa-miR-1343-5p | 0 | 0 | 2.422287 |
| hsa-miR-4498 | 0 | 2.259698 | 0 |
| novel_157 | 0 | 354.7726 | 0 |
| hsa-miR-3194-3p | 0 | 2.259698 | 0 |
| hsa-miR-181b-2-3p | 0 | 9.038792 | 4.844574 |
| hsa-miR-4521 | 0 | 0 | 2.422287 |
| hsa-miR-6881-5p | 0 | 0 | 2.422287 |
| hsa-miR-589-3p | 0 | 2.259698 | 0 |
| hsa-miR-183-3p | 0 | 2.259698 | 0 |
| hsa-miR-6726-3p | 0 | 2.259698 | 0 |
| hsa-miR-491-3p | 0 | 0 | 2.422287 |
| hsa-miR-4803 | 0 | 0 | 2.422287 |
| hsa-miR-500b-3p | 0 | 2.259698 | 0 |
| hsa-miR-34a-3p | 0 | 4.519396 | 4.844574 |
| hsa-miR-3928-3p | 0 | 6.779094 | 2.422287 |
| hsa-miR-4746-5p | 0 | 2.259698 | 0 |
| hsa-miR-5088-3p | 0 | 0 | 2.422287 |
| hsa-miR-514a-3p | 0 | 0 | 7.266861 |
| hsa-miR-1909-3p | 0 | 2.259698 | 0 |
| hsa-miR-6886-5p | 0 | 0 | 2.422287 |
| hsa-miR-4745-3p | 0 | 0 | 2.422287 |
| hsa-miR-3127-3p | 0 | 0 | 2.422287 |
| hsa-miR-4683 | 0 | 2.259698 | 0 |
| hsa-miR-7705 | 0 | 2.259698 | 0 |
| hsa-miR-103a-2-5p | 0 | 9.038792 | 2.422287 |
| hsa-miR-3142 | 0 | 4.519396 | 0 |
| novel_180 | 0 | 38.41487 | 21.80058 |
| novel_190 | 0 | 45.19396 | 46.02345 |
| novel_189 | 0 | 36.15517 | 29.06744 |
| novel_183 | 0 | 24.85668 | 41.17888 |
| hsa-miR-2467-5p | 0 | 2.259698 | 0 |
| novel_188 | 0 | 1129.849 | 1496.973 |
| novel_186 | 0 | 433.862 | 700.0409 |
| novel_158 | 0 | 171.7371 | 106.5806 |
| novel_169 | 0 | 24.85668 | 33.91202 |
| hsa-miR-6816-3p | 0 | 0 | 2.422287 |
| hsa-miR-4470 | 0 | 0 | 2.422287 |
