# Supplementary figures and images for: Umbilical mesenchymal stem cell-derived exosomes facilitate spinal cord functional recovery through the miR-199a-3p/145-5p-mediated NGF/TrkA signaling pathway in rats
Source: Stem Cell Res Ther. 2021 Feb 12;12:117. doi: 10.1186/s13287-021-02148-5 (PMC7879635; doi:10.1186/s13287-021-02148-5)

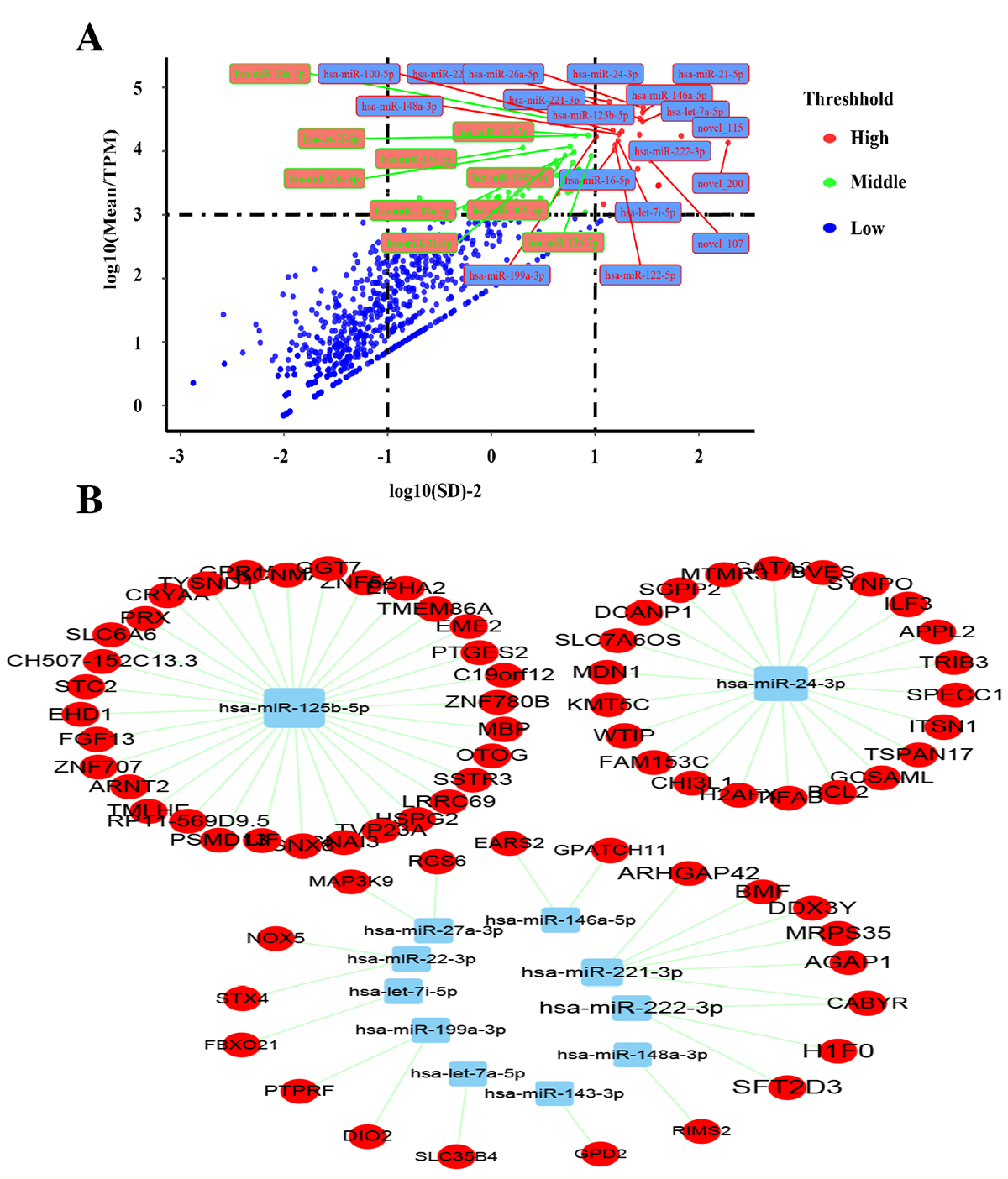

Supplement: Supplementary file 7 — Additional file 7. Bioinformatic analysis of miRNAs. (A) The linear relationship between lgSD-2 and lgTPM. (B) Predicted target genes of the top 20 miRNAs (plus miR-145-5p) in miRanda. TPM, transcripts per million; SD, standard difference. [file 13287_2021_2148_MOESM7_ESM.tif]

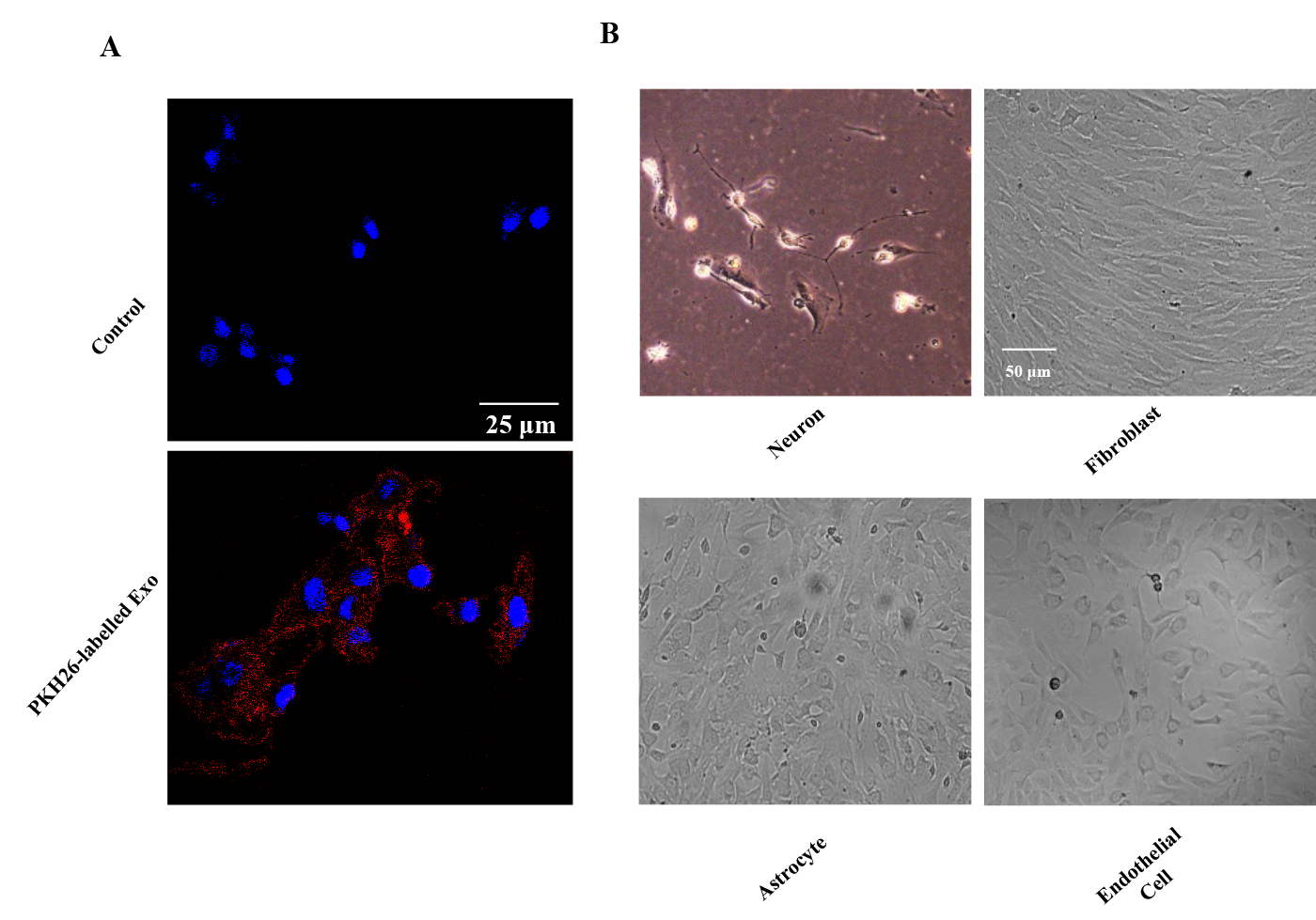

Supplement: Supplementary file 8 — Additional file 8. Images of MSC-Exo taken up by PC12 cells and miR-199a-3p and miR-145-5p expression profiles in different cells. (A) Representative images of MSC-Exo taken up by PC12 cells. Scale bar=25 μm. (B) The morphology of primary neurons, astrocytes, endothelial cells and meningeal fibroblasts visualized by an inverted fluorescence microscope. Scale bar=50 μm. MSC-Exo, MSC-derived exosomes; QRT-PCR, quantitative real-time polymerase chain reaction. [file 13287_2021_2148_MOESM8_ESM.tif]

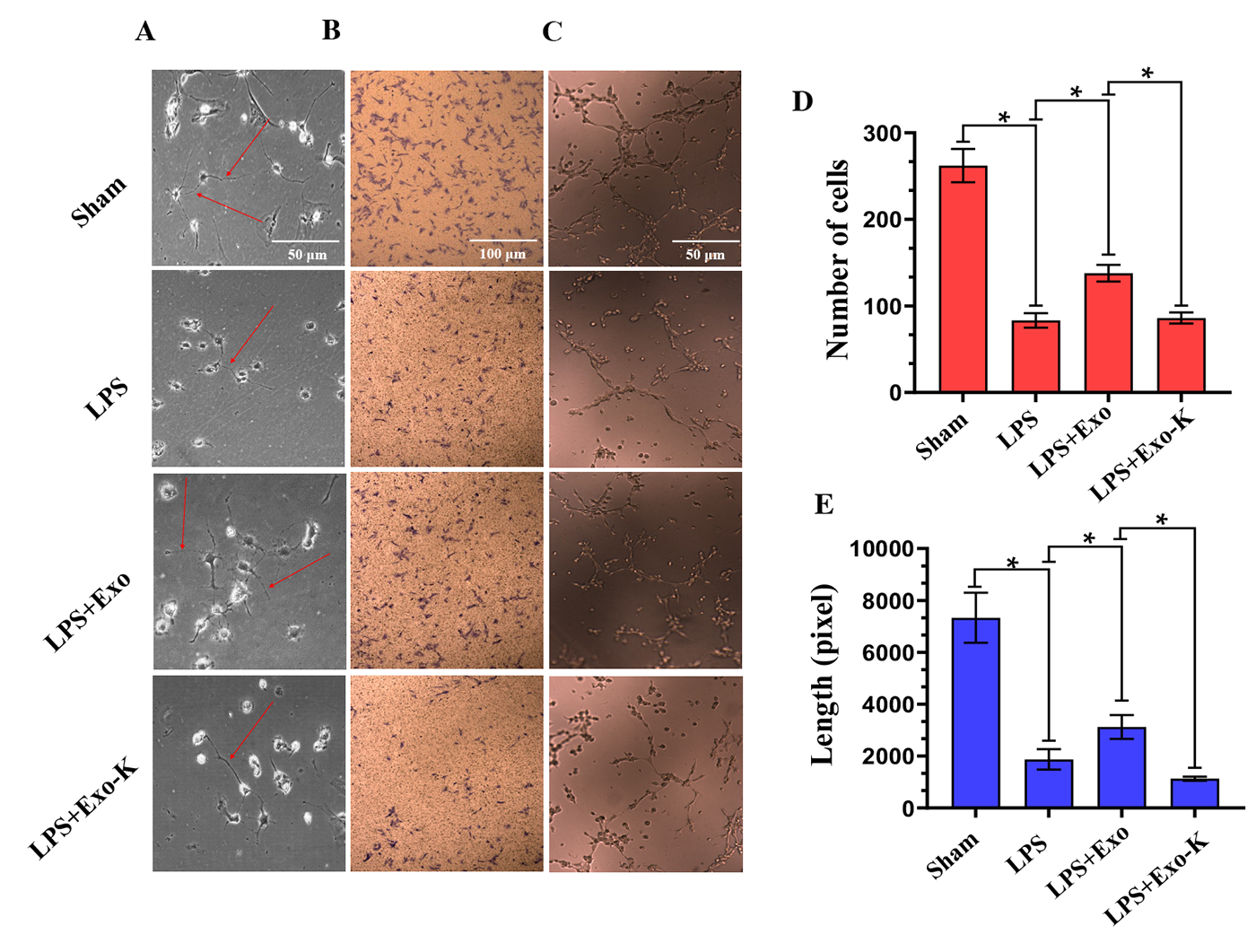

Supplement: Supplementary file 9 — Additional file 9. The effects of exosomal miR-199a-3p/miR-145-5p on endothelial cell migration, tube formation, and neurite outgrowth of primary neurons in vitro. (A) Representative images of neurite outgrowth (red arrow) in LPS-stimulated primary neurons pretreated with Exo and Exo-K. Scale bar=50 μm. (B) Representative images of cell migration in LPS-stimulated endothelial cells pretreated with Exo and Exo-K (n=3). Scale bar=50 μm. (C) Representative images of tube formation in LPS-stimulated endothelial cells pretreated with Exo and Exo-K (n=3). Scale bar=50 μm. (D) Quantitative analysis of migrated cells in (B) and (E) the length of the tubes in (C). Data are represented as mean ± SD. LPS, lipopolysaccharide; MSC, mesenchymal stem cell; MSC-Exo, MSC-derived exosomes; Exo-K, MSC-Exo with the inhibition of miRNAs. [file 13287_2021_2148_MOESM9_ESM.tif]

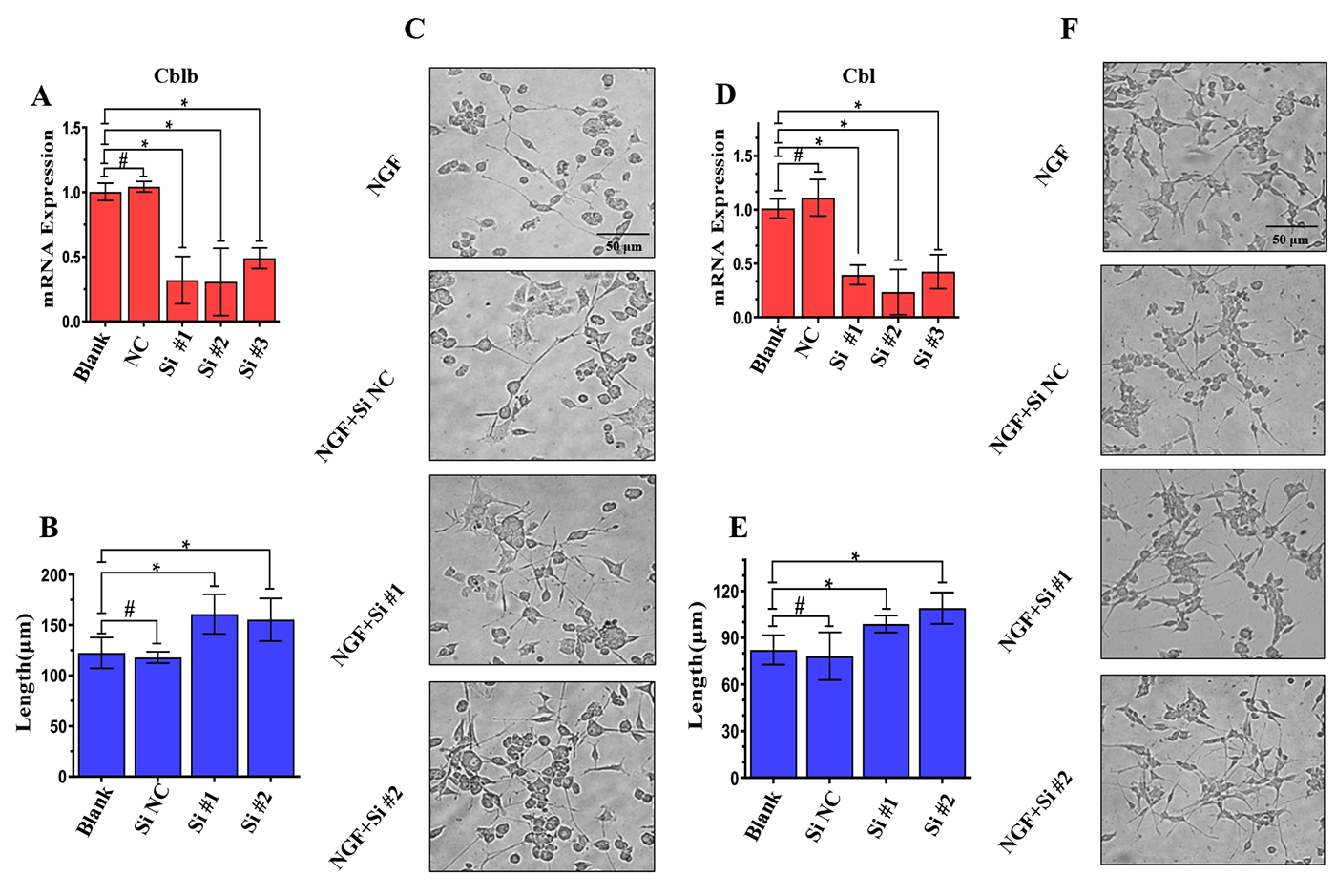

Supplement: Supplementary file 10 — Additional file 10. Knockdown of Cblb and Cbl increased NGF-induced neurite outgrowth in PC12 cells. Cblb mRNA level in nontransfected PC12 cells and PC12 cells transfected with scramble sequence, Cblb SiRNA #1, SiRNA #2, and SiRNA #3 as detected by QRT-PCR (A). Data are represented as mean ± SD. Quantitative analysis (B) and representative images (C) of cell neurite growth in NGF-stimulated PC12 cells transfected with scramble sequence, Cblb SiRNA #1 and SiRNA #2. Scale bar=50 μm. Data are represented as mean ± SD. Cbl mRNA level in nontransfected PC12 cells and PC12 cells transfected with scramble sequence, Cbl SiRNA #1, SiRNA #2, and SiRNA #3 as detected by QRT-PCR (D). Data are represented as mean ± SD. Quantitative analysis (E) and representative images (F) of cell neurite growth in NGF-stimulated PC12 cells transfected with scramble sequence, Cblb SiRNA #1 and SiRNA #2. Scale bar=50 μm. Data are represented as mean ± SD. NGF, neuronal growth factor; QRT-PCR, quantitative real-time polymerase chain reaction. [file 13287_2021_2148_MOESM10_ESM.tif]

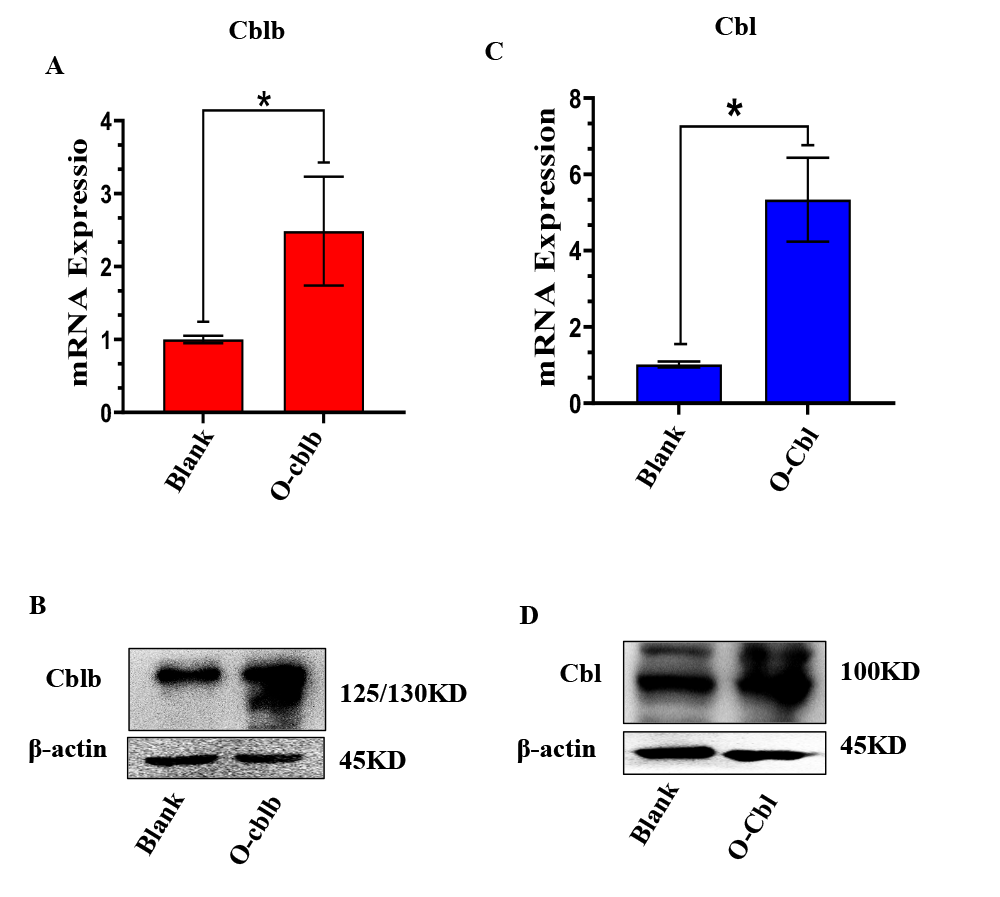

Supplement: Supplementary file 11 — Additional file 11. Overexpression of Cblb and Cbl in PC12 cells. (A) Quantitative analysis of Cblb mRNA expression by QRT-PCR (B) and representative western blot images of Cblb. (C) Quantitative analysis of Cbl mRNA expression by QRT-PCR (B) and representative western blot images of Cbl. Data are represented as mean ± SD. QRT-PCR, quantitative real-time polymerase chain reaction.; EGFP; enhanced green fluorescent protein. [file 13287_2021_2148_MOESM11_ESM.tif]

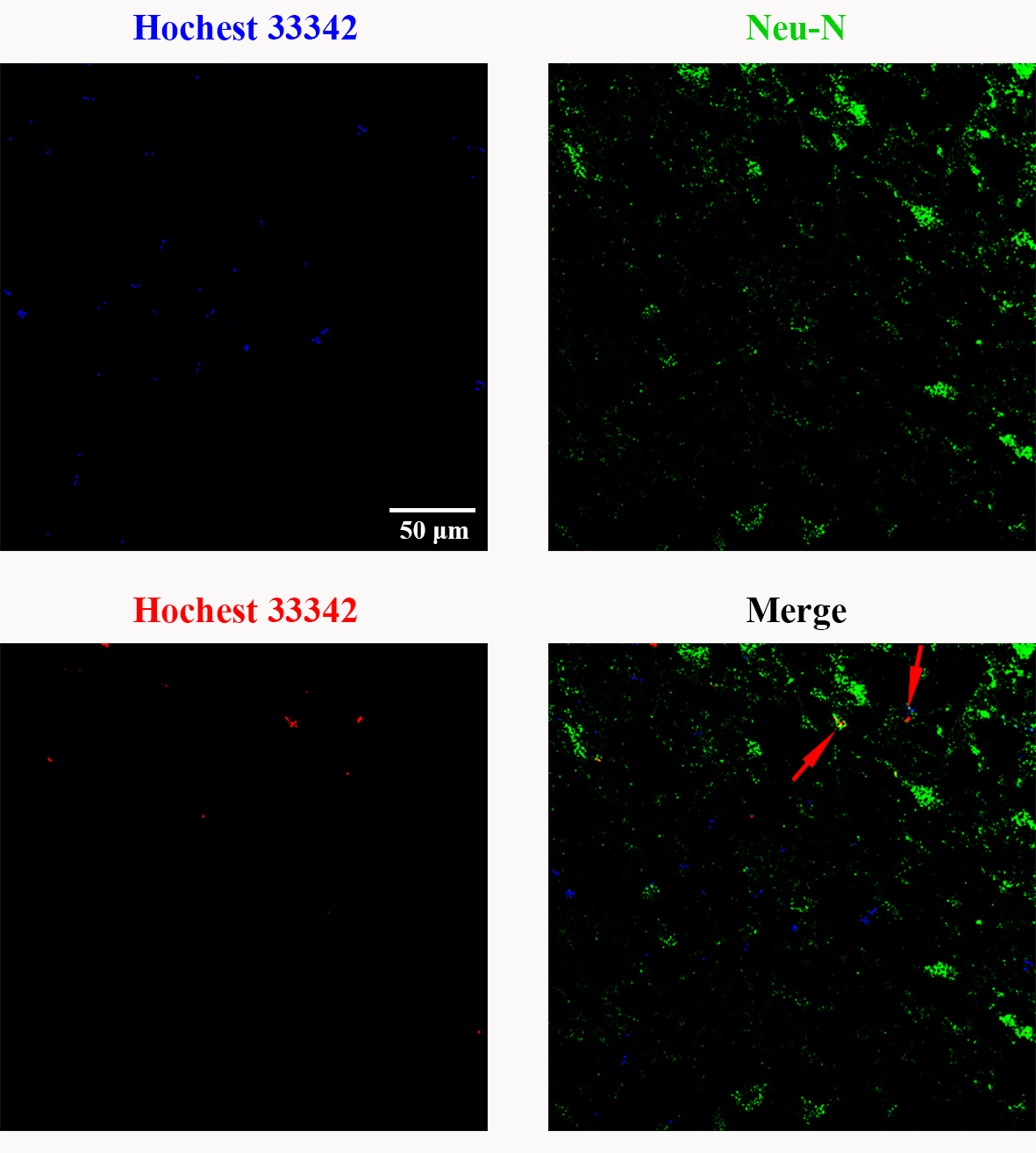

Supplement: Supplementary file 12 — Additional file 12. Track of PKH26-labelled exosomes in vivo by confocal microscope. The nucleus (blue), Neu-N (green) and PHK26 (red and indicated by the red arrow) were stained to identify the uptake of exosomes by the injured neurons. [file 13287_2021_2148_MOESM12_ESM.tif]

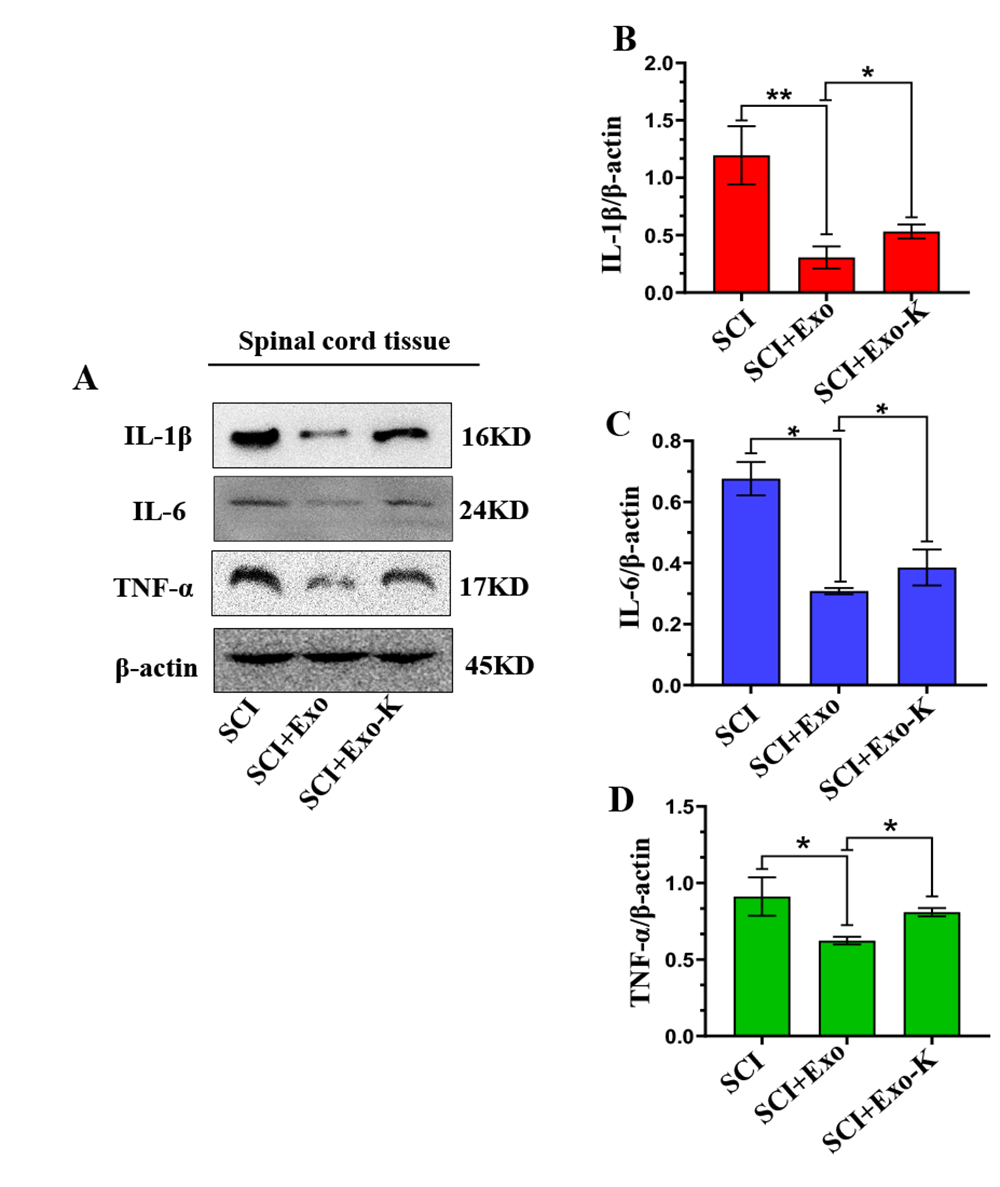

Supplement: Supplementary file 13 — Additional file 13. Exosomal 199a-3p/145-5p strongly reduced inflammation levels. Representative western blot images (A) and quantitative analysis of IL-1 (B), IL-6 (C) and TNF-α (D). Data are represented as mean ± SD. [file 13287_2021_2148_MOESM13_ESM.tif]
